# Low-dose in vivo protection and neutralization across SARS-CoV-2 variants by monoclonal antibody combinations

**DOI:** 10.1038/s41590-021-01068-z

**Published:** 2021-10-29

**Authors:** Vincent Dussupt, Rajeshwer S. Sankhala, Letzibeth Mendez-Rivera, Samantha M. Townsley, Fabian Schmidt, Lindsay Wieczorek, Kerri G. Lal, Gina C. Donofrio, Ursula Tran, Nathaniel D. Jackson, Weam I. Zaky, Michelle Zemil, Sarah R. Tritsch, Wei-Hung Chen, Elizabeth J. Martinez, Aslaa Ahmed, Misook Choe, William C. Chang, Agnes Hajduczki, Ningbo Jian, Caroline E. Peterson, Phyllis A. Rees, Magdalena Rutkowska, Bonnie M. Slike, Christopher N. Selverian, Isabella Swafford, I-Ting Teng, Paul V. Thomas, Tongqing Zhou, Clayton J. Smith, Jeffrey R. Currier, Peter D. Kwong, Morgane Rolland, Edgar Davidson, Benjamin J. Doranz, Christopher N. Mores, Theodora Hatziioannou, William W. Reiley, Paul D. Bieniasz, Dominic Paquin-Proulx, Gregory D. Gromowski, Victoria R. Polonis, Nelson L. Michael, Kayvon Modjarrad, M. Gordon Joyce, Shelly J. Krebs

**Affiliations:** 1 Emerging Infectious Diseases Branch, Walter Reed Army Institute of Research, Silver Spring, MD USA; 2 U.S. Military HIV Research Program, Walter Reed Army Institute of Research, Silver Spring, MD USA; 3 Henry M. Jackson Foundation for the Advancement of Military Medicine, Bethesda, MD USA; 4 Laboratory of Retrovirology, The Rockefeller University, New York, NY USA; 5 Milken Institute School of Public Health, The George Washington University, Washington, DC USA; 6 Viral Diseases Branch, Walter Reed Army Institute of Research, Silver Spring, MD USA; 7 Integral Molecular, Philadelphia, PA USA; 8 Vaccine Research Center, NIAID, NIH, Bethesda, MD USA; 9 NCI, NIH, Bethesda, MD USA; 10 Trudeau Institute, Saranac Lake, NY USA; 11 Howard Hughes Medical Institute, The Rockefeller University, New York, NY USA; 12 Center of Infectious Disease Research, Walter Reed Army Institute of Research, Silver Spring, MD USA

**Keywords:** Antimicrobial responses, Antibodies, Viral infection

## Abstract

Prevention of viral escape and increased coverage against severe acute respiratory syndrome coronavirus 2 (SARS-CoV-2) variants of concern require therapeutic monoclonal antibodies (mAbs) targeting multiple sites of vulnerability on the coronavirus spike glycoprotein. Here we identify several potent neutralizing antibodies directed against either the N-terminal domain (NTD) or the receptor-binding domain (RBD) of the spike protein. Administered in combinations, these mAbs provided low-dose protection against SARS-CoV-2 infection in the K18-human angiotensin-converting enzyme 2 mouse model, using both neutralization and Fc effector antibody functions. The RBD mAb WRAIR-2125, which targets residue F486 through a unique heavy-chain and light-chain pairing, demonstrated potent neutralizing activity against all major SARS-CoV-2 variants of concern. In combination with NTD and other RBD mAbs, WRAIR-2125 also prevented viral escape. These data demonstrate that NTD/RBD mAb combinations confer potent protection, likely leveraging complementary mechanisms of viral inactivation and clearance.

## Abstract (web summary)

Krebs and colleagues identify multiple mAbs that recognize either the RBD or the NTD of SARS-CoV-2 spike protein that have potent cross-neutralizing activities against variants of concern. Combinatorial mAb cocktails have complementary effects on viral neutralization and Fc effector functions and can protect against SARS-CoV-2 escape mutants.

## Main

Since its emergence in late 2019, SARS-CoV-2, the causative agent of the coronavirus disease originating in 2019 (COVID-19), has precipitated a pandemic of unprecedented proportion. The isolation of mAbs that provide prophylactic protection or therapeutic benefit against the circulating SARS-CoV-2 viral variants have been a major goal toward curbing the pandemic. Therapeutic neutralizing mAbs that have received emergency use authorization or are currently in clinical development target the RBD of SARS-CoV-2 spike (S) protein^1–5^. Within the SARS-CoV-2 S glycoprotein, the RBD plays a critical role by engaging the human angiotensin-converting enzyme 2 (ACE2) receptor in the lungs, initiating viral entry and infection^6^. A second class of SARS-CoV-2 neutralizing antibodies target the NTD of the S protein, a domain located at the periphery of the S trimer. To date, all NTD-directed neutralizing antibodies target a single antigenic supersite within this subdomain^7,8^. Regardless of these advances, several widely circulating viral variants of concern (VOCs) have been able to evade neutralization by mAb therapies^9,10^, highlighting the urgent need for the development of broad therapeutic countermeasures.

In this study, we identify several potent neutralizing RBD-directed and NTD-directed mAbs using a nanoparticle displaying the S glycoprotein to capture SARS-CoV-2-specific B cells. Combinations of RBD and NTD mAbs offered enhanced in vivo protection by leveraging beneficial attributes specific to each class. Combinations of mAbs targeting NTD/RBD prevented viral escape in vitro and offered broader coverage over currently circulating VOCs, including the Delta strain.

## Results

### Isolation of potent SARS-CoV-2 neutralizing antibodies

**Fig. 1:**
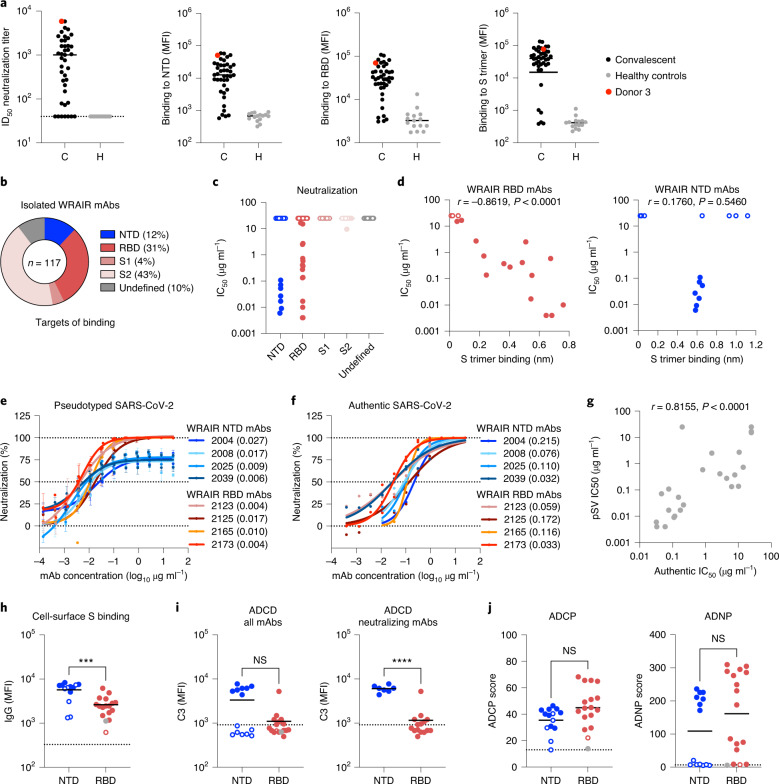
Isolation of SARS-CoV-2 neutralizing antibodies from a convalescent donor. **a**, Plasma neutralization against SARS-CoV-2 and from convalescent (C) and healthy (H) donors. Convalescent Donor 3, highlighted in red, was selected for B cell sorting based on high plasma neutralization against IL1/2020 and high-magnitude binding antibodies to the NTD, RBD and S trimer, as measured in a multiplex bead-based assay. Bars indicate the median value. MFI, mean fluorescence intensity. **b**, The percentage of isolated mAbs binding to the subdomains of S protein. **c**, Neutralization potency of isolated WRAIR mAbs segregated by subdomain binding specificity. Shown are the mean IC_50_ values (µg ml^−1^) from the SARS-CoV-2 (IL1/2020) pseudotyped assay, calculated from three independent experiments. **d**, Correlation between neutralization potency (IC_50_) for NTD-directed (right, *n* = 14 *xy* pairs) and RBD-directed (left, *n* = 18 *xy* pairs) mAbs and their respective binding magnitude to the SARS-CoV-2 stabilized S trimer, obtained from a single experiment. A significant (inverse) correlation was only observed for the WRAIR RBD-directed mAbs. Spearman *r* values are indicated above each graph with *P* values (two-tailed). **e**,**f**, Neutralization curves of the most potent NTD-directed and RBD-directed neutralizing antibodies as measured in the pseudotyped (**e**) and authentic (**f**) SARS-CoV-2 assays, using strains IL1/2020 and INMI1/2020, respectively, which share an identical S sequence. Plotted are the mean ± s.e.m. from three (**e**) or two (**f**) independent experiments. The IC_50_ value (µg ml^−1^) for each mAb is indicated in parentheses and calculated using a five-parameter regression analysis. **g**, Correlation between the pSV and authentic virus assays, *n* = 24 *xy* pairs. The Spearman *r* value and *P* value (two-tailed) are indicated above the graph. **h**, NTD and RBD WRAIR mAb binding to cell-surface-expressed S protein using 293F cells as measured by flow cytometry. Black lines indicate the mean value and asterisks represent significance by two-tailed Mann–Whitney *t*-test; *P* = 0.0009. The dotted line indicates the positivity threshold. **i**,**j**, Assessment of NTD and RBD mAbs recruitment of Fc-mediated complement (ADCD; **i**) and phagocytic activities (ADCP and ADNP; **j**). ADCD was measured using an S-expressing 293F cell line, whereas phagocytic activities were determined using the stabilized S trimer. Black horizontal lines indicate the mean value and asterisks represent significance by two-tailed Mann–Whitney *t*-test; *P* < 0.0001. The dotted line indicates the positivity threshold. **h**–**j**, Shown are representative data (*n* = 2) from a single experiment. In **c**, **d** and **h**–**j**, neutralizing and non-neutralizing mAbs are in closed and open circles, respectively, while the control RBD mAb CR3022 is shown as a gray closed circle in **h**–**j**. Source data

**Extended Data Fig. 1:**
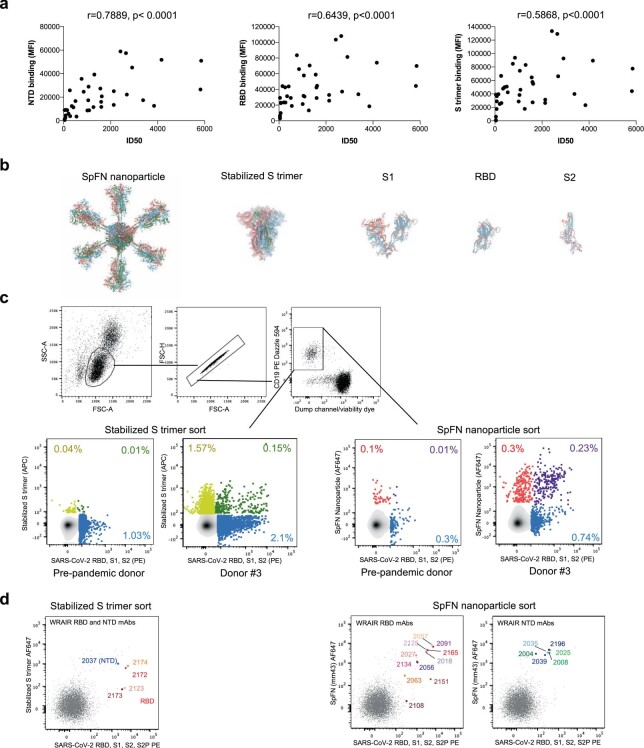
Serology of convalescent donors and sorting strategy to isolate SARS-CoV-2 reactive B cells. **a**, Correlation of plasma binding magnitude and neutralization potency of convalescent COVID-19 donors. Spearman *r* and *P* (two-tailed) values are indicated above each graph. *n* = 41 xy pairs for each of the test. **b**, Antigens used to sort SARS-CoV-2 positive B cells. Two sorting strategies were performed using either a stabilized S trimer (HexaPro) or a multivalent Spike ferritin nanoparticle (SpFN) displaying 8 Spike trimers, to isolate antibodies targeting conformational or quaternary epitopes, in addition to S1, RBD and S2 SARS-CoV-2 subdomain antigens. **c**, Gating strategy used to sort antigen^+^ B cells with the percentage of SARS-CoV-2 antigen positive B cells from a pre-pandemic donor and SARS-CoV-2 convalescent Donor #3 obtained with the two complementary sorting approaches, using the stabilized S trimer (left) or SpFN (right). **d**, Individual CD19 + SARS-CoV-2 reactive B cells encoding SARS-CoV-2 neutralizing mAbs indicated in the flow cytometry plots for the stabilized S trimer (left) and SpFN nanoparticle (right) sorts. Source data

**Extended Data Fig. 2:**
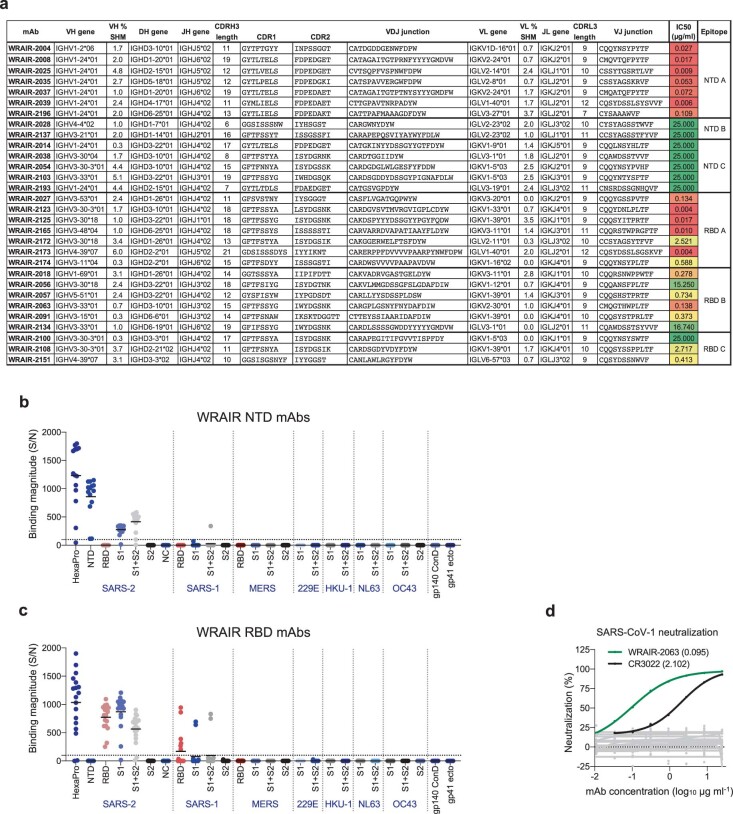
Genetics and cross-reactivity of characterized WRAIR NTD and RBD mAbs. **a**, Gene assignment was performed with IgBlast using Kabat numbering. SHM, somatic hypermutation; CDR, complementarity determining region; VDJ, variable, diversity and joining genes. IC50 values from the pseudovirus neutralization assay, as well as competition groups are indicated. Only two clonally related mAbs were identified, WRAIR-2008 and -2037. **b, c**, Binding of WRAIR NTD (b) and RBD (c) mAbs to a panel of 26 human CoV and HIV control (gp140 and gp41) antigens, assessed in a multiplex bead-based assay. Binding magnitude is expressed as signal/noise (S/N) ratio, with noise calculated from an HIV mAb antibody control (MZ4). S/N ≥ 10 (dotted line) were considered positive based on negative control binding. **d**, SARS-CoV-1 (Sino1-11) neutralization activity in a pseudotyped assay. Data are mean from two independent experiments. WRAIR-2063 is indicated in green with the IC50 (µg ml^-1^) indicated in parenthesis. The CR3022 positive control is indicated in black. Other WRAIR RBD and NTD mAbs that did not neutralize SARS-CoV-1 are indicated in grey. Source data

**Extended Data Fig. 3:**
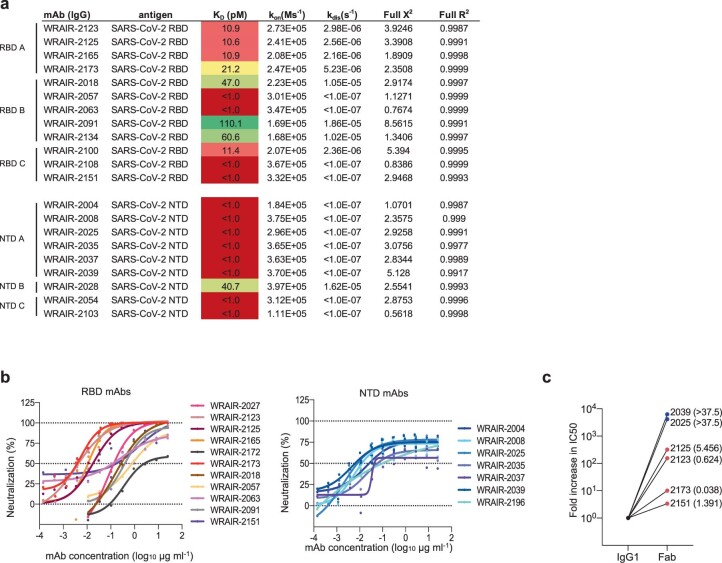
Binding affinity and functional characteristics of WRAIR mAbs against SARS-CoV-2 IL1/2020. **a**, Binding affinity constants of WRAIR RBD and NTD mAbs measured against their respective domains using BLI. At least 4 curves from a dilution series were used to calculate the equilibrium dissociation constant (KD) using a 1:1 binding model. KD values are colored dark red to green from low to high. **b**, Neutralization curves of WRAIR RBD (left) and NTD (right) mAbs obtained in the pSV assay. Shown are mean from at least 2 independent experiments. Error bars were omitted for clarity. **c**, Comparison of neutralization activities between IgG1 and Fabs. Potent NTD- (blue) or RBD- (red) neutralizing antibodies were assessed for neutralization in the PSV assay either as IgG1 or Fabs. Shown is the fold increase in IC50 observed with the Fab versions of each mAb compared to its IgG1 counterpart. IC50 (μg ml^-1^) values obtained with Fabs are indicated in parentheses. Source data

Convalescent plasma samples of 56 SARS-CoV-2-infected human donors, who had mild to moderate symptoms, were screened for neutralization potency. Among them, Donor 3 demonstrated potent neutralization and high antibody binding to NTD, RBD and the prefusion stabilized S trimer^11^ (S trimer hereafter; Fig. 1a). Binding to NTD, RBD and the S trimer strongly correlated with plasma neutralization of pseudotyped SARS-CoV-2 virions (pseudotyped lentivirus (pSV); Extended Data Fig. 1a). While previous isolation efforts utilized RBD or the S trimer as probes^12–17^, we sought to obtain a comprehensive understanding of neutralizing antibodies elicited by SARS-CoV-2 infection by using peripheral blood mononuclear cells (PBMCs) from Donor 3 in two independent sorting strategies to isolate SARS-CoV-2-specific CD19^+^ B cells with a broad range of specificities. The first sorting strategy used a combination of SARS-CoV-2 (USA-IL1/2020) probes that included the S trimer, RBD and S1 and S2 subunits. In the second sort, the S trimer was replaced by a multivalent spike ferritin nanoparticle (SpFN) displaying eight S trimers (Extended Data Fig. 1b), a vaccine candidate currently in a phase I clinical trial (NCT04784767)^18,19^. SpFN was used to mimic the SARS-CoV-2 virus with the desire to isolate mAbs targeting potential conformational or quaternary epitopes. The two sorting strategies revealed complementary profiles in their ability to bind to antigen-specific B cells using flow cytometry, with a high overall frequency of SpFN- and S trimer-specific B cells (Extended Data Fig. 1c). The majority of potent NTD-directed neutralizing mAbs were isolated from the SpFN sort, whereas RBD neutralizing antibodies were obtained from both sorting approaches (Extended Data Fig. 1d). In aggregate, 213 antibody heavy-chain and light-chain pairs were recovered from both sorting strategies and sequenced from single-cell SARS-CoV-2-positive B cells. Antibodies were produced as human IgG1 in Expi293F cells and screened as cell culture supernatants for binding and neutralization. A total of 117 mAbs were subsequently purified and tested for binding to SARS-CoV-2 subdomains and for neutralization using an S protein pSV neutralization assay. The majority of the mAbs bound to S2, which may have been a result of the sorting strategy, followed by RBD and NTD, based on binding antibody assays (Fig. 1b). As potent neutralization activity was only observed for RBD-directed and NTD-directed antibodies (Fig. 1c), we focused our efforts on these two classes of antibodies. RBD-directed and NTD-directed mAbs exhibited low levels of somatic hypermutation and a wide range of complementarity determining region (CDR) H3 lengths. Each mAb belonged to individual clonal families, except for two related NTD mAbs, Walter Reed Army Institute of Research (WRAIR)-2008 and WRAIR-2037 (Extended Data Fig. 2a). Binding cross-reactivity across human alpha and beta coronaviruses demonstrated that isolated NTD mAbs were SARS-CoV-2 specific, whereas a few RBD mAbs cross-reacted with SARS-CoV-1 (Extended Data Fig. 2b,c). Of these, WRAIR-2063 was able to potently neutralize SARS-CoV-1 with a 50% inhibitory concentration (IC_50_) of 95 ng ml^−1^ (Extended Data Fig. 2d). RBD mAbs demonstrated neutralization potency ranging from subnanomolar to micromolar concentrations, whereas NTD mAbs presented a dichotomous profile being either strongly neutralizing or non-neutralizing (Fig. 1c). RBD mAbs revealed a strong correlation between neutralization potency and binding magnitude to the S trimer (Fig. 1d). In contrast, binding to the S trimer did not correlate with neutralization by NTD-targeting mAbs. All NTD neutralizing mAbs displayed intermediate binding to the S trimer, whereas binding responses observed with non-neutralizing NTD mAbs were either high or absent, revealing three distinct binding profiles (Fig. 1d). Irrespective of their neutralization activity, NTD and RBD mAbs strongly bound to their respective S subdomains with dissociation constants (*K*_D_) within or below the picomolar range (Extended Data Fig. 3a).

We next compared the neutralization potency of these mAbs in pSV and authentic SARS-CoV-2 virus neutralization assays. NTD mAbs displayed potent neutralization in both assays, with a notable difference: neutralization curves plateaued at around 75% neutralization in the pSV assay, as previously observed^7,20^, while the same NTD mAbs achieved 100% neutralization of authentic SARS-CoV-2 (Fig. 1e,f). The two neutralization assays closely correlated with one another (Fig. 1g), in agreement with previous studies^21^. In the pSV neutralization assay, all NTD-targeting neutralizing mAbs demonstrated IC_50_ values below 100 ng ml^−1^, with WRAIR-2039 and WRAIR-2025 being the most potent at 6 and 9 ng ml^−1^, respectively (Fig. 1e,f and Extended Data Fig. 3b). RBD mAbs achieved 100% neutralization in both assay types with a wider range of potency spanning several orders of magnitude (Fig. 1e,f and Extended Data Fig. 3b). WRAIR-2173 and WRAIR-2123 were the most potent with identical IC_50_ values of 4 ng ml^−1^, followed by WRAIR-2165 (10 ng ml^−1^) and WRAIR-2125 (17 ng ml^−1^). When tested as Fabs, WRAIR NTD mAbs no longer neutralized the pSV, suggesting that bivalent binding and/or the presence of the Fc domain in the IgG1 format is required for pSV neutralization (Extended Data Fig. 3c). Fab versions of RBD mAbs, such as WRAIR-2173 and WRAIR-2151, retained most of their potency but others, like WRAIR-2123 and WRAIR-2125, had markedly reduced activity by over two orders of magnitude, possibly reflecting differences in their mechanism of action (Extended Data Fig. 3c).

**Extended Data Fig. 4:**
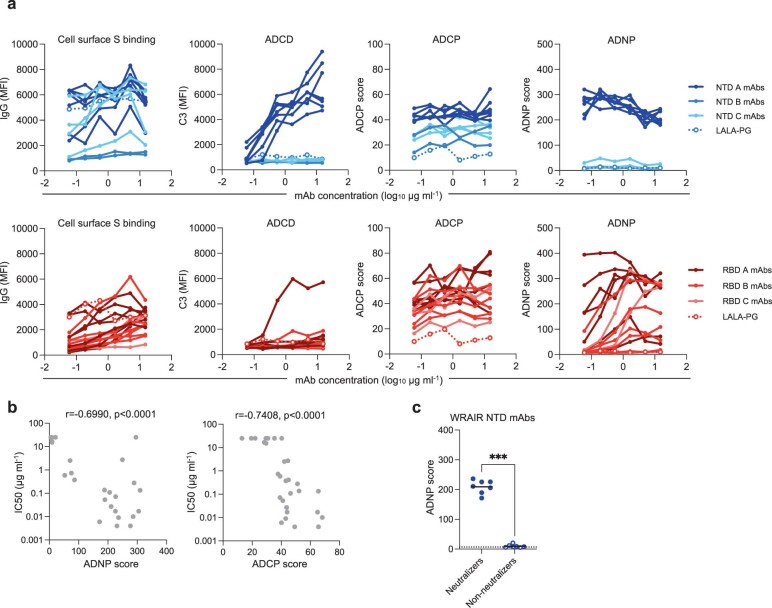
Fc effector functions of WRAIR mAbs against SARS-CoV-2 IL1/2020. **a**,Titrations of WRAIR NTD (blue) and RBD (red) mAbs in the Fc effector function assays. Antibodies are colored according to their competition groups. Fc effector functions were measured twice and shown are data from a single representative experiment. A Fc mutant control (LALA-PG) is shown for reference with open circle and dotted line. **b**, Correlation between neutralization activity and phagocytic activities for all mAbs. Spearman *r* and *P* (two-tailed) values are indicated above the graph, *n* = 31 xy pairs. **c**, Difference in ADNP score between NTD neutralizing and non-neutralizing mAbs. Black horizontal line indicates mean value and asterisks represent significance by two-tailed Mann–Whitney t-test, *P* = 0.0006, *n* = 7. Dotted line indicates positivity threshold. Source data

In addition to neutralization activity, Fc effector functions have also been shown to play an important functional role in protection against SARS-CoV-2 in vivo^22–25^. Therefore, we investigated the ability of the NTD and RBD mAbs, all expressed as IgG1, to promote Fc effector functions (Extended Data Fig. 4a). NTD-targeting mAbs, inclusive of non-neutralizing mAbs, were significantly better than RBD-targeting mAbs at mediating opsonization of cells expressing S protein at their surface (Fig. 1h), a prerequisite for any Fc effector activities against virus-infected cells. Antibody-dependent complement deposition (ADCD) was only observed for the neutralizing NTD mAbs, indicating that non-neutralizing NTD epitopes may not be compatible with complement recruitment (Fig. 1i). Interestingly, only one RBD mAb (WRAIR-2165) was able to recruit complement at a similar magnitude compared to the WRAIR NTD mAbs, and as such, neutralizing NTD mAbs displayed significantly higher ADCD activity than RBD neutralizing mAbs (Fig. 1i). Determination of phagocytic activities with monocytes (antibody-dependent cellular phagocytosis (ADCP)) and neutrophils (antibody-dependent neutrophil phagocytosis (ADNP)) using the S trimer demonstrated that both NTD and RBD neutralizing mAbs performed equally well, with higher scores significantly correlating with neutralization activity (Fig. 1j and Extended Data Fig. 4b). However, neutralizing NTD mAbs were significantly better at mediating ADNP compared to non-neutralizing mAbs (Extended Data Fig. 4c). Collectively, we identified potent neutralizing antibodies directed against the SARS-CoV-2 NTD and RBD that mediate multiple Fc effector functions, with the NTD mAbs demonstrating the unique ability to promote complement deposition.

### Epitope characterization of NTD-targeting mAbs

**Fig. 2:**
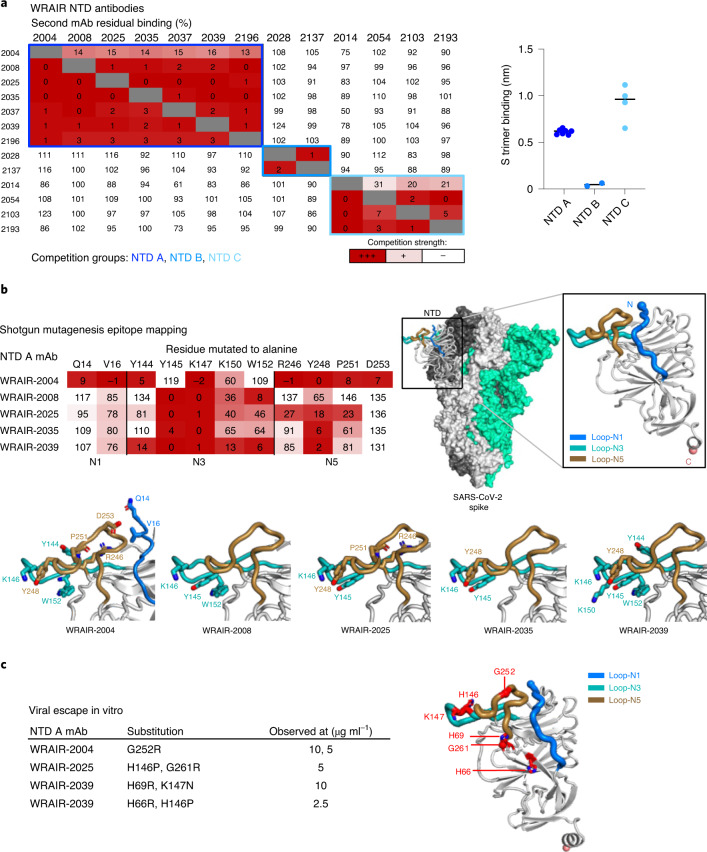
Epitope mapping and structural characterization of WRAIR NTD mAbs. **a**, Left, epitope binning of NTD-directed mAbs via a BLI-based competition assay. Values represent the percentage of residual binding of the indicated second antibody after saturation of the antigen (NTD domain) with the indicated first antibody. Shading from dark to light red indicates competition strength ranging from strong (0–25%), to intermediate (25–50%) to none (>50%). Competition groups are indicated by boxes in shades of blue. Right, binding responses of NTD-directed mAbs, segregated by competition group, to the stabilized S trimer measured by BLI. **b**, Left, epitope mapping of NTD A mAbs using a shotgun mutagenesis platform. The heat map shows the percentage of binding to NTD mutants, harboring a single change to alanine at the indicated position, relative to wild type. Right, the NTD (residues 14–303) is shown in the context of the SARS-CoV-2 trimer (PDB 6ZGE) with loops N1, N3 and N5 colored in blue, teal and gold, respectively. Bottom, key binding residues are shown on the NTD structure with side chains shown in stick representation. **c**, Left, residues identified in the viral escape assay in the presence of NTD antibodies at the indicated concentrations. Right, the same residues are shown in stick representation and labeled in bold red on the NTD structure. Source data

We next used a biolayer interferometry (BLI) competition binding assay as a first step to delineate the antigenic sites targeted by these mAbs (Fig. 2a). WRAIR NTD mAbs fell into three distinct groups; all neutralizing antibodies clustered into one group (NTD A), while non-neutralizing antibodies clustered into two groups (NTD B and C) that differed by their ability to bind the S trimer. While NTD C mAbs bound strongly to the S trimer, NTD B mAbs only interacted with the isolated NTD domain, likely recognizing a cryptic epitope hidden in the ‘closed’ prefusion S trimer (Fig. 2a). Notably, many NTD A neutralizing antibodies used an IGHV1-24 heavy chain (Extended Data Fig. 2a), similarly to previous mAbs isolated in several convalescent donors^8,20,26,27^ such as 4A8 (ref. ^26^), 1-87 (ref. ^8^) and CM25 (ref. ^27^; Supplementary Table 1). Secondly, to further characterize the epitopes targeted by the NTD neutralizing antibodies, we mapped epitopes using a shotgun mutagenesis platform, which measures loss of binding. Despite variations in their antibody CDR H3 lengths and sequences (Extended Data Fig. 2a), binding of the VH1-24-derived NTD neutralizing mAbs was affected by mutations in the N3 (Y145, K147) and/or N5 (R246, Y248) loops within the previously characterized NTD antigenic supersite^7,8^. The epitope of NTD mAb WRAIR-2004 (VH1-2 gene) was more extensive with the inclusion of residues in N1 (Q14, V16), in addition to residues in N3 (Y144, K147) and N5 (R246, Y248, P251 and D253; Fig. 2b). These results were further confirmed by growing a recombinant vesicular stomatitis virus (VSV), encoding SARS-CoV-2 S protein (rVSV/SARS-CoV-2/GFP virus), in vitro in the presence of NTD neutralizing antibodies (Fig. 2c). All selected viral variants had substitutions in N3 and/or N5 loops at the same position or in the vicinity of the residues identified by the shotgun mutagenesis approach (Fig. 2c). Overall, we identified three non-competing groups of NTD-directed antibodies, with NTD A mAbs demonstrating high affinity and neutralization potency.

### Structural determination of receptor-binding domain-targeting antibodies

**Fig. 3:**
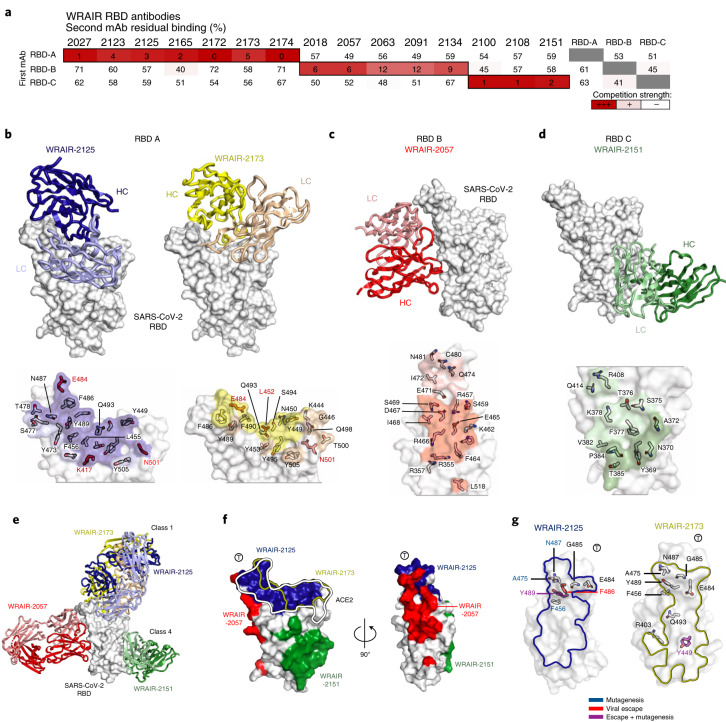
Structure and epitope determination of SARS-CoV-2 RBD-targeting mAbs. **a**, Epitope binning of RBD-directed mAbs via a BLI-based competition assay. Values represent the percentage of residual binding of the indicated second antibody after saturation of the antigen (RBD molecule) with the indicated first antibody. Shading from dark to light red indicates competition strength ranging from strong (0–25%), to intermediate (25–50%), to lack thereof (>50%). Competition groups are indicated by black boxes. Control antibodies RBD A, RBD B and RBD C were CC12.1, CC12.16 and CR3022, respectively. **b**–**d**, Top, representative crystal structures of RBD-targeting antibodies for WRAIR RBD groups A, B and C are shown. RBD A mAbs, WRAIR-2125 (dark blue) and WRAIR-2173 (yellow) target the ACE2 binding site. RBD B mAb, WRAIR-2057 (red) recognizes a novel epitope on the ‘side’ of the RBD distal from the ACE2 binding site centered on residue E465. RBD C mAb, WRAIR-2151 (dark green) targets a CR3022-like site on the RBD. Bottom, epitope footprints of respective antibodies are shown on the surface of the RBD and colored based on the antibody heavy-chain and light-chain colors. RBD contacting residues are shown as sticks, with residues seen in VOCs highlighted in bold red. **e**, Structures of WRAIR RBD A, B and C antibodies are shown on a single RBD molecule to highlight the different recognition modes. **f**, RBD A, B and C epitopes are shown on the RBD surface with the ACE2 binding interface highlighted by the black/white line. **g**, Epitope mapping of WRAIR-2125 and WRAIR-2173 contact residues identified in the shotgun mutagenesis (blue) and viral escape experiments (red), or both (purple) are shown in stick representation.

**Extended Data Fig. 5:**
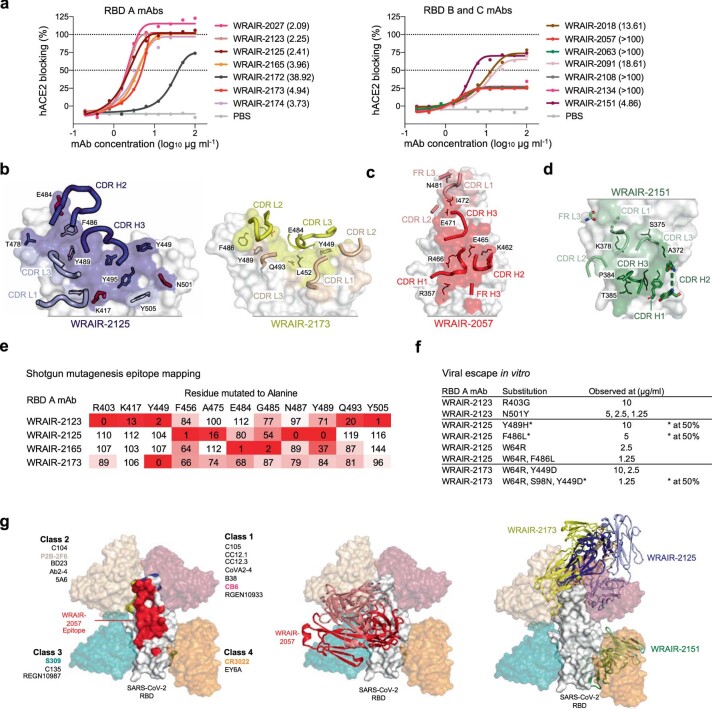
Epitope mapping and structural characterization WRAIR RBD mAbs. **a**, ACE2 blocking activity of WRAIR RBD mAbs. WRAIR RBD A (left) and B and C (right) mAbs were assessed for their ability to block ACE2 binding to SARS-CoV-2 RBD in a BLI-based assay. The half maximal effective concentration (EC50) in µg ml^-1^ is indicated in parentheses. **b-d**, Details of RBD A (WRAIR-2125 and -2173) (b), RBD B (WRAIR-2057) (c) and RBD C (WRAIR-2151) (d) epitopes. Antibody residues are shown in stick representation and RBD residues are shown in line representation. Contributing heavy and light chain CDRs are shown and labelled. CDR loops are designated using the Kabat numbering system. **e**, Epitope mapping of RBD A mAbs using a shotgun mutagenesis platform. Heat map shows % binding to RBD mutants, harboring a single change to Alanine at the indicated position, relative to wild-type. **f**, Residues identified in the viral escape assay in presence of RBD antibodies at the indicated concentrations. Asterisks indicate mutations found only in half of the sequences obtained. **g**, Structures of WRAIR RBD A, B and C antibodies are overlaid on previously reported antibodies (representing frequently observed SARS-CoV-2 epitopes). Left and middle panel: WRAIR-2057 antibody and epitope is shown in red on the RBD surface in the context of previously reported antibody classes. Right panel: WRAIR-2125, WRAIR-2173 and WRAIR-2151 are shown with representative Class 1, 2, 3 and 4 mAbs. Source data

To gain insights into the epitopes targeted by the RBD neutralizing mAbs, we conducted similar binding antibody competitions as described above. Based on their competition with previously described mAbs CC12.1, CC12.16 and CR3022 (refs. ^13,28^), WRAIR RBD neutralizing mAbs segregated into three distinct groups: RBD A, B and C, respectively (Fig. 3a). The most potent neutralizing mAbs belonged to the RBD A group, which encompassed previously defined RBD mAb classes 1 and 2 that compete strongly with ACE2 (ref. ^29^; Extended Data Fig. 5a). To understand the structural basis of RBD recognition, crystal structures of representative group A, B and C mAbs in complex with the RBD were determined (Fig. 3b–e, Extended Data Fig. 5b–d and Supplementary Tables 2 and 3). Crystal structures of group A potent neutralizing antibodies WRAIR-2125 and WRAIR-2173 in complex with the SARS-CoV-2 RBD were analyzed to a final resolution of 3.77 Å and 2.2 Å, respectively. Both group A mAbs target the ACE2 binding site with overlapping, but distinct epitopes (Fig. 3b,e,f and Extended Data Fig. 5b).

WRAIR-2173 forms extensive interactions across the entire length of the ACE2 receptor-binding region, whereas WRAIR-2125 is focused to one side and engages fewer RBD residues (Fig. 3b and Extended Data Fig. 5b). The WRAIR-2125 epitope buries >890 Å^2^ of surface area with heavy and light chains contributing 65% and 35% of total buried surface area (BSA), respectively (Supplementary Table 3), and is primarily based on CDR H2–3 and CDR L1 and L3 interactions (Supplementary Tables 2 and 3). This includes antibody hydrophobic CDR H2–3 residues V50, Y58, Y99, P100G and CDR L1–3 residues Y32, Y92 and I93, which stack against a hydrophobic patch of the RBD-ACE2 binding site (L455, F456, Y473, F486 and Y489).

**Extended Data Fig. 6:**
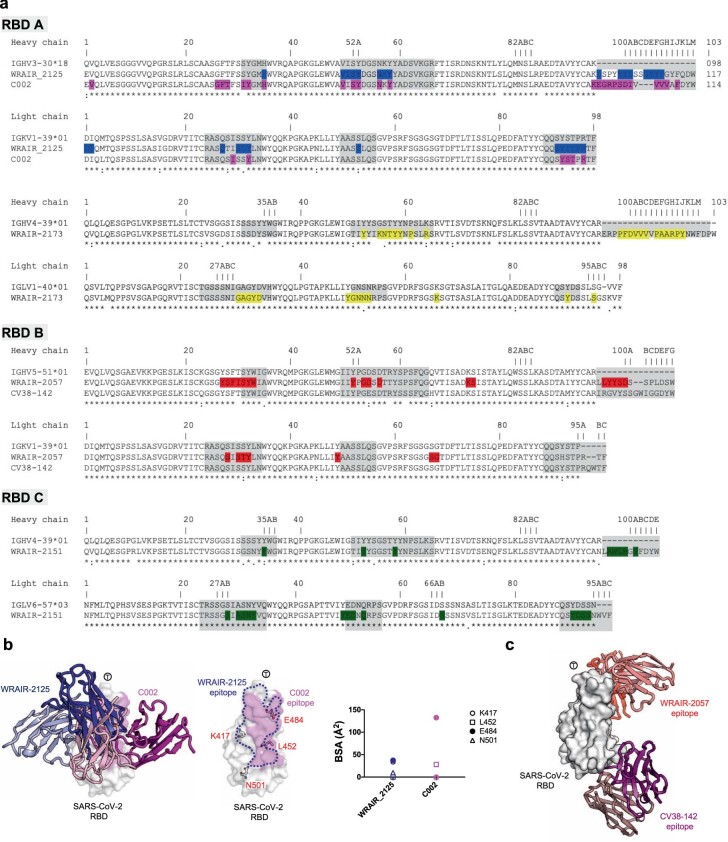
Structure and sequence alignment. **a**, Sequence alignment of RBD A, B and C WRAIR mAbs with their precursor germline genes. Antibody residue numbers are labelled on the top and CDRs are shaded gray. All antibody residue numbering and CDR loops are designated using the Kabat numbering system. Antibody residues that interact with RBD are colored as antibody structures in Fig. 2. Symbols *,:, and. denote identical, similar and less similar residues, respectively. Previously published antibodies, C002 and CV38-142, with identical germline-encoded genes as WRAIR-2125 and WRAIR-2057, respectively, are also aligned. **b**, *Left panel*: C002 structure (magenta) is overlaid onto the WRAIR-2125 (dark blue) structure. *Middle panel*: Frequently occurring SARS-CoV-2 VOC residues are shown as sticks on the RBD surface with WRAIR-2125 and C002 epitopes highlighted in dark blue and magenta colors, respectively. *Right panel*: Buried surface area (BSA) for VOC residues, related to the mAbs WRAIR-2125 and C002 are shown as dot plots. T symbol is used to designate the “tip” of the RBD molecule. **c**, MAb CV38-142 structure (purple) is overlaid onto the WRAIR-2057-RBD complex structure (red). Source data

The WRAIR-2173 mAb epitope is >900 Å^2^ with heavy and light chains contributing ~65% and 35% of total BSA, respectively (Supplementary Table 3). WRAIR-2173 recognition of SARS-CoV-2 RBD is also based primarily on CDR H2–3 and CDR L1–3 (Fig. 3b and Extended Data Fig. 5b). The CDR H2 and H3 loops cover ~200 Å^2^ and >400 Å^2^ of the RBD interface, respectively (Supplementary Tables 2 and 3). CDR H2 residues K55, N56, T57 and Y58 interact with RBD residues 483–486, while CDR H3 recognition involves extensive hydrophobic contacts using CDR H3 residues P98–Y100J to interact with RBD residues K444, Y449, N450, L452 and Q493–Y495. Both WRAIR-2125 and WRAIR-2173 form strong interactions with RBD F486 overlapping with RBD-ACE2 contact residues (Fig. 3b,f,g). Shotgun mutagenesis-based epitope mapping experiments confirmed the ACE2 binding site as the target for RBD A antibodies and identified F486, N487 and Y489 as critical residues of the WRAIR-2125 epitope, while WRAIR-2173 binding was only moderately affected by mutations at these sites (Fig. 3f and Extended Data Fig. 5e). Viral escape experiments also identified p.Phe486Leu and p.Tyr489His as escape mutations for WRAIR-2125 and p.Tyr449Asp for WRAIR-2173, each in agreement with the structural and epitope mapping data (Fig. 3f and Extended Data Fig. 5f). Based on the structural superimposition with representative antibodies from previously defined classes, WRAIR-2125 and WRAIR-2173 were grouped into class 1 mAbs (Extended Data Fig. 5g). While WRAIR-2125 shares heavy-chain and light-chain germline genes with a previously reported mAb, C002 (ref. ^29^), both mAbs have dissimilar CDR H3 sequences and target different epitopes on the RBD (Extended Data Fig. 6a,b and Supplementary Table 1).

Representative group B mAb WRAIR-2057 binds to a unique epitope located on the ‘side’ of the RBD molecule, distal from the ACE2 binding site (Fig. 3c,e,f and Extended Data Fig. 5c,g). Antibodies that target the RBD B epitope have been seen in other convalescent donor samples^13,30^, but to our knowledge, this is the first high-resolution structure reported. The epitope covers a BSA of 855 Å^2^ with heavy and light chains contributing 72.5% and 27.5% of total BSA, respectively (Supplementary Table 3). WRAIR-2057 recognition of SARS-CoV-2 RBD is primarily based on CDR H1–3 and CDR L1 (Fig. 3c, Extended Data Fig. 5c and Supplementary Tables 2 and 3). Heavy-chain interactions form a total of six hydrogen bonds and three salt-bridges with the RBD along with a set of CDR H1 and H3 hydrophobic residues involved in major contacts (Supplementary Tables 2 and 3), while light-chain contacts are primarily mediated by CDR L1 and L2. WRAIR-2057 shares heavy-chain (IGVH5–51) and light-chain (IGKV1–39) germline gene usage with SARS-CoV-2 mAb CV38–142 (ref. ^31^). However, these antibodies have distinct nonoverlapping epitopes (Extended Data Fig. 6a,c).

Representative group C mAb WRAIR-2151 binds to the previously defined CR3022 epitope on the RBD^28,32^ (Fig. 3d–f and Extended Data Fig. 5g), burying >670 Å^2^ with heavy and light chains contributing 37.5% and 62.5% of the total BSA, respectively (Supplementary Table 3). WRAIR-2151 recognition of SARS-CoV-2 RBD is primarily based on CDR H2–3 and CDR L1–3 (Fig. 3d, Extended Data Fig. 5d and Supplementary Tables 2 and 3). Overall contacts are mediated by both hydrophobic and hydrophilic residues (Fig. 3d, Extended Data Fig. 6a and Supplementary Table 3). In summary, we determined the molecular determinants of four RBD-directed neutralizing antibodies belonging to three different classes each with distinct features that bind to SARS-CoV-2.

### Low-dose in vivo prophylactic protection conferred by NTD and RBD monoclonal antibodies

**Fig. 4:**
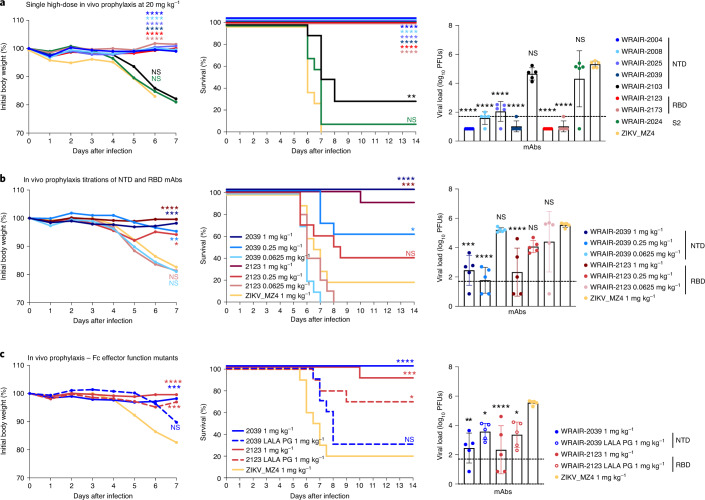
WRAIR monoclonal antibodies offer low-dose prophylactic protection in the K18-hACE2 mouse model. **a**–**c**, Antibodies were infused intravenously at a single high dose of 400 µg (20 mg per kg body weight; **a**) or low doses of 2 µg (1 mg per kg body weight) and lower (**b** and **c**) into groups of mice (*n* = 15 per group). Mice were challenged intranasally 24 h later with 1.25 × 10^4^ viral particles (1.25 × 10^4^ PFUs) of SARS-CoV-2 (WA1/2020). SARS-CoV-2 viral loads in lung tissue were measured 2 d after challenge in a subset of animals (*n* = 5 per group) by plaque assay. Bars indicate the mean group value with standard deviation. The remaining mice (*n* = 10 per group) were assessed daily for weight and clinical symptoms. **c**, Assessment of Fc effector functions on animal protection for NTD and RBD antibodies. Wild-type and LALA-PG versions of mAb WRAIR-2039 (NTD) and WRAIR-2123 (RBD) were compared at 20 µg (1 mg per kg body weight). For weight loss and viral load in lungs, asterisks indicate significance compared to the ZIKV_MZ4 mAb isotype control group, by one-way analysis of variance (ANOVA) with Dunnett’s multiple-comparisons test. Survival curves were compared individually to the isotype control using a Mantel–Cox log-rank test. For all tests, ****P < 0.0001, ***P < 0.001, **P < 0.01, *P < 0.5; NS, not significant (*P* > 0.5). Source data

We next determined whether WRAIR NTD and RBD mAbs could confer protection in vivo with a series of experiments using the K18-hACE2 transgenic SARS-CoV-2 mouse model^33,34^. To assess protection provided by prophylaxis, mAbs were infused intravenously 24 h before intranasal challenge with an 80% lethal dose of SARS-CoV-2 (1.25 × 10^4^ plaque-forming units (PFUs) WA1/2020). Using a high dose of 400 µg (20 mg per kg body weight) of either NTD or RBD neutralizing mAbs provided complete protection (Fig. 4a). In contrast, S2-targeting mAb WRAIR-2024 and NTD non-neutralizing mAb WRAIR-2103 did not prevent infection or death at the same concentration of 20 mg per kg body weight (Fig. 4a), suggesting that targeting neutralization epitopes is important for in vivo protection.

To determine the minimal protective dose for prophylactic protection, we next titrated the passively administered potent neutralizing mAbs WRAIR-2039 (NTD) and WRAIR-2123 (RBD) until protection was lost (Fig. 4b). Remarkably, a 5-µg (0.25 mg per kg body weight) dose of the NTD mAb WRAIR-2039 used alone was sufficient to suppress viral replication in the lungs, confirming the high potency of NTD-directed mAbs in vivo, while the lowest dose where protection was observed was 1 mg per kg body weight for RBD mAb WRAIR-2123 (Fig. 4b).

**Extended Data Fig. 7:**
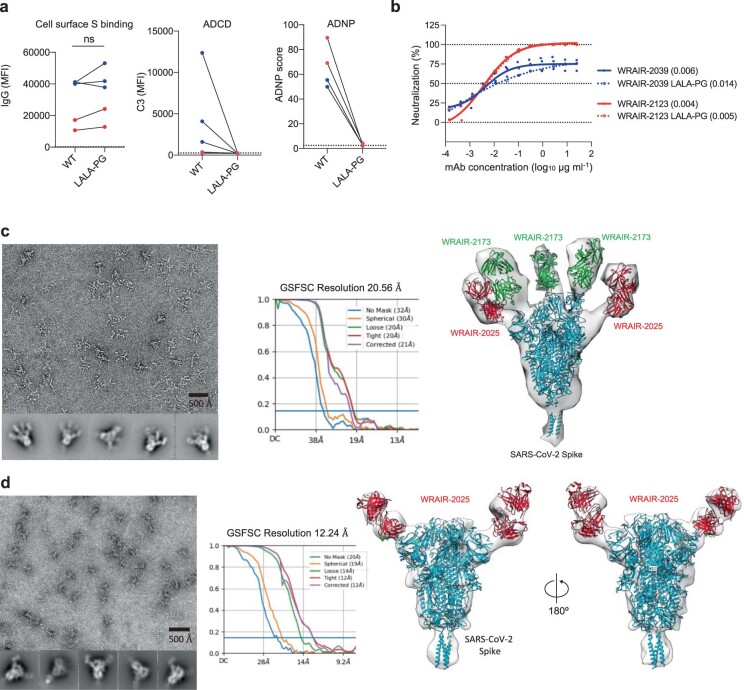
Characterization of LALA-PG mutant mAbs and negative stain electron microscopy of Spike-Fab complexes. **a**, Characterization of the LALA-PG mutant forms of the NTD mAbs WRAIR-2039, 2025 and 2004 (in blue) and RBD mAbs WRAIR-2173 and 2123 (in red) in cell surface S binding (left), ADCD (middle) and ADNP (right) assays. Non-significant differences in binding to cell surface S was observed between WT and mutants in a Wilcoxon matched-pairs signed rank test, P = 0.3125. LALA-PG mutants did not show any activity in the ADCD and ADNP assays. **b**, Neutralization curves of WT and LALA-PG versions of WRAIR-2039 (NTD) and -2123 (RBD) mAbs obtained in the pSV assay. Shown are mean ± SD from at least 2 independent experiments. IC50 (µg ml^-1^) are indicated in parentheses in the legend. **c**, EM analysis of WRAIR-2173 and WRAIR-2025 Fabs in complex with SARS-CoV-2 spike (S-2P) trimer. *Left panel*: Raw image (top) and two-dimensional class particle averages (bottom, 5 averages shown). The black bars represent 500 Å. *Middle panel*: Gold-standard FSC curves for the EM 3D reconstruction. *Right panel*: Negative-stain 3D reconstruction of SARS-CoV-2 spike and Fab complex. The structural model of the SARS-CoV-2 trimer (PDB 6X2B) in complex with WRAIR-2173 and WRAIR-2025 Fabs is shown in ribbon representation while negative-stain electron density map is shown as a gray transparent surface (also shown in Fig. 5b). A featureless and unbiased (lowpass filter: 100Å) 3D model of SARS-CoV-2 spike trimer (PDB: 6VXX) and 12,574 particles were used to perform the 3D reconstruction from a single experiment. **d**, EM analysis of WRAIR-2025 Fab in complex with SARS-CoV-2 spike (S-2P) trimer. *Left panel*: Raw image (top) and two-dimensional class averages (bottom, 5 averages shown) of Fab-Spike particles. The black scale bars represent 500 Å. *Middle panel*: Gold-standard FSC curves for the EM 3D reconstruction. *Right panel*: Negative-stain 3D reconstruction of SARS-CoV-2 Spike and Fab complex. The structural model of the SARS-CoV-2 trimer (PDB 6VXX) and WRAIR-2025 Fab is shown in ribbon representation while negative-stain electron density map is shown as a gray transparent surface. A feature less and unbiased (lowpass filter: 100Å) 3D model of SARS-CoV-2 spike trimer (PDB: 6VXX) and 3,364 particles were used to perform the 3D reconstruction from a single experiment. Source data

Finally, because NTD and RBD mAbs displayed a wide range of Fc effector functions in vitro, with NTD neutralizing mAbs unique to their class in demonstrating high ADCD activity (Fig. 1h), we sought to examine whether the in vivo potency observed could be explained by engagement of Fc effector functions. RBD mAb WRAIR-2123 and NTD mAb WRAIR-2039 were modified to harbor a triple mutation (LALA-PG)^35^ ablating all Fc effector functions, while maintaining binding to cell-surface-expressed S protein and potent neutralization (Extended Data Fig. 7a,b). When tested in vivo for prophylactic protection following passive transfer, the RBD mAb WRAIR-2123 LALA-PG mutant revealed partial protection at the 20-µg (1 mg per kg body weight) dose, with over half of the animals surviving infection (Fig. 4c). The requirement of Fc effector functions for in vivo protection was more pronounced for the NTD WRAIR-2039 LALA-PG mAb, where most of the animals succumbed to infection by day 8, with modest suppression of viral load in the lungs (Fig. 4c).

### Evaluation of NTD-targeting and RBD-targeting monoclonal antibody combinations

**Fig. 5:**
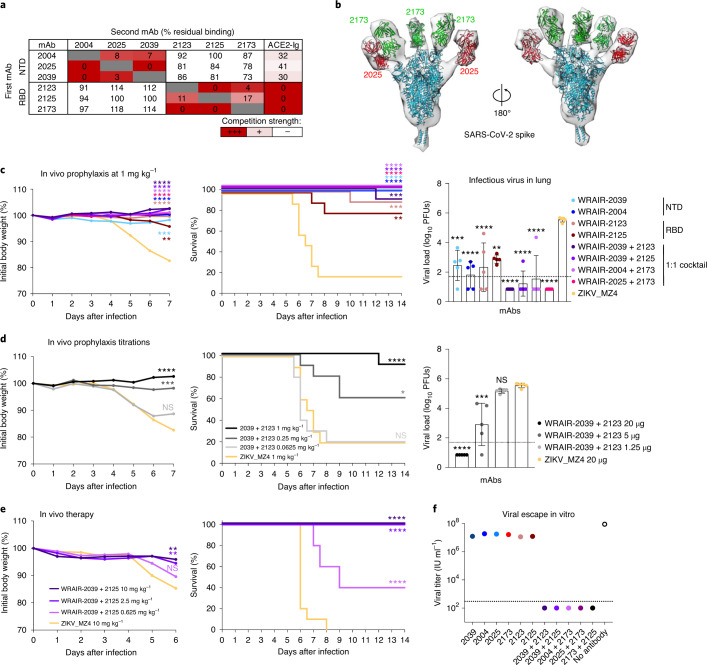
NTD/RBD monoclonal antibody combinations offer low-dose in vivo protection and a higher genetic barrier for viral escape. **a**, Binding competition to the stabilized trimer as described in Fig. 2a. **b**, Negative-stain three-dimensional reconstruction of SARS-CoV-2 spike in complex with WRAIR-2025 (NTD) and WRAIR-2173 (RBD) Fabs. **c**,**d**, Prophylactic treatment in the K18-hACE2 SARS-CoV-2 mouse model. Antibodies were infused intravenously at a dose of 20 µg (1 mg per kg body weight) or titrated as single mAbs or combinations (1:1 ratio) into groups of mice (*n* = 15 per group). Mice were challenged intranasally 24 h later with 1.25 × 10^4^ viral particles (1.25 × 10^4^ PFUs) of SARS-CoV-2 (WA1/2020). SARS-CoV-2 viral loads in lung tissue were measured 2 d after challenge in a subset of animals (*n* = 5 per group) by plaque assay. Bars indicate the mean group value with standard deviation. The remaining mice (*n* = 10 per group) were assessed daily for weight and clinical symptoms. **e**, Therapeutic treatment in the K18-hACE2 SARS-CoV-2 mouse model. Antibodies were infused intravenously at the indicated dose 24 h after challenge, performed as indicated above. Mice (*n* = 15 per group) were assessed daily for weight and clinical symptoms. **d**,**e**, For weight loss and viral load in lungs, asterisks indicate significance compared to the ZIKV_MZ4 isotype control group by one-way ANOVA with Dunnett’s multiple-comparisons test. Survival curves were compared individually to the isotype control using a Mantel–Cox log-rank test. For all analysis, ****P < 0.0001, ***P < 0.001, **P < 0.01, *P < 0.5; NS, *P* > 0.5. **f**, Viral titers of a replicative rVSV/SARS-CoV-2/GFP virus obtained after two passages in the presence of single mAbs or combinations. Plotted are the means from two independent experiments. Source data

**Extended Data Fig. 8:**
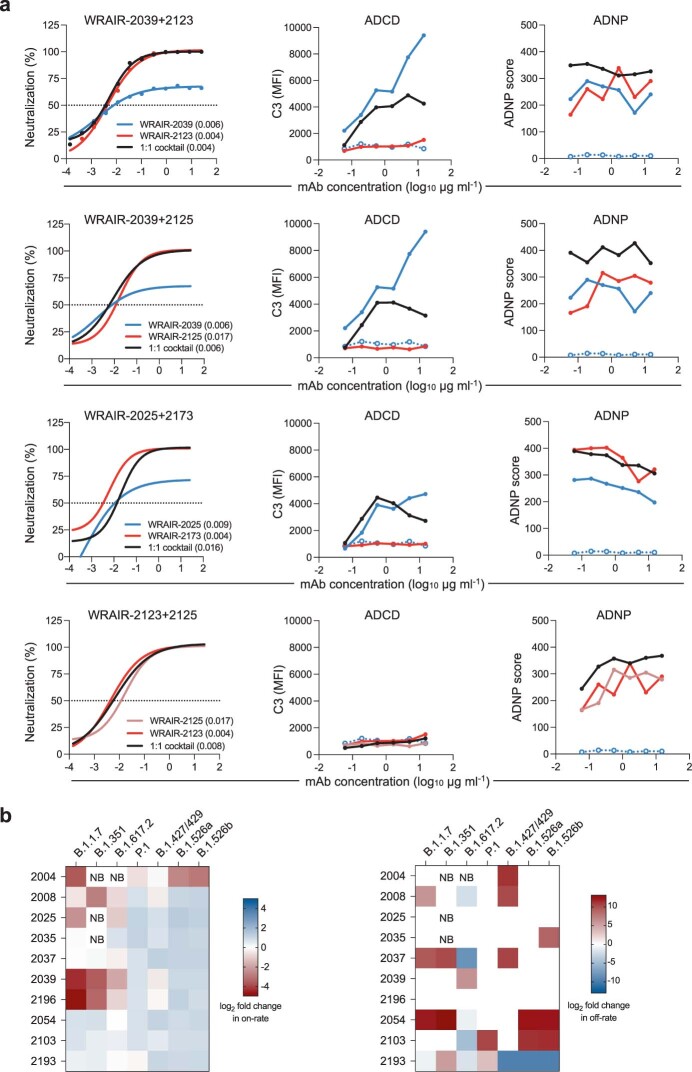
Functional activities of WRAIR mAb combinations against SARS-CoV-2 IL1/2020 and characterization of variant binding. **a**, Functional activities of NTD/RBD and RBD/RBD mAb combinations. WRAIR-2039, -2025 (NTD) and WRAIR-2123, -2125 and -2173 (RBD) were mixed in a 1:1 ratio as indicated and the cocktail was assessed for neutralization (pSV assay) and Fc effector functions. Single mAbs or cocktail were tested at the same final antibody concentration. IC50 values (µg ml^-1^) obtained in the pSV assay from 2 independent experiments are indicated in parentheses. ADCD and ADNP activities were measured twice and shown are data from a single representative experiment. The WRAIR-2039 LALA-PG negative control is shown as open circles and dotted blue line. **b**, Effect of mutations present in VOC and VOI on the binding on-rate and off-rate of NTD-directed mAbs. Binding on-rates (left) and off-rates (right) to stabilized S trimer (S-2P) harboring mutations present in the indicated variant was assessed by BLI. On- and off-rates were obtained by fitting the binding curves of mAbs at a single concentration of 200 nM using a 1:1 binding model. Heat-map shows the log_2_ fold change relative to a WA1/2020 D614G S-2P spike protein with negatively impacted mAbs (by either a decrease in on-rate or increase in off-rate) represented in shades of red. Shades of blue represent mAb with improved binding kinetics, compared to the WT. NB indicate absence of value due to lack of binding. Source data

Combining mAbs that target different sites on the surface of the viral spike could offer advantages by mitigating the risk for viral escape^36,37^. To assess the compatibility of our potent neutralizing NTD and RBD mAbs, we performed competition experiments using the S trimer. Pre-incubation of the S trimer with the neutralizing NTD mAbs did not prevent subsequent binding of the RBD mAbs (Fig. 5a). Similarly, the S trimer pre-bound with group A RBD mAbs retained full capacity to engage NTD mAbs (Fig. 5a), indicating binding of both classes of mAbs simultaneously. Modest inhibition of ACE2 binding was observed with the NTD mAbs (Fig. 5a), likely due to steric hindrance through their light-chain and/or Fc domains, as previously described for Middle East respiratory syndrome coronavirus (MERS-CoV) NTD-targeting antibodies^38^. In contrast, all neutralizing RBD A mAbs fully blocked ACE2 binding (Fig. 5a). Negative-stain electron microscopy (EM) analysis further confirmed that NTD mAb WRAIR-2025 and RBD mAb WRAIR-2173 engaged the S trimer concomitantly, albeit with different stoichiometry (Fig. 5b). Two copies of the NTD mAb WRAIR-2025 were observed for most of the complexes, whereas all three RBD subdomains of the S trimer were occupied by RBD mAb WRAIR-2173 (Fig. 5b and Extended Data Fig. 7c,d). To verify that combining NTD and RBD mAbs would not abrogate their neutralization activity, we tested several combinations of the most potent WRAIR mAbs in pSV neutralization assays, formulated at a 1:1 ratio so that each mAb in the combination contributes to 50% of the total concentration of the respective mAbs tested alone. Combinations of NTD and RBD mAbs demonstrated potent pSV neutralization, and on par with neutralization potency of the single mAbs alone, indicating that NTD and RBD mAbs retained their full activity when mixed (Extended Data Fig. 8a). Likewise, combinations of NTD and RBD mAbs retained strong Fc effector functions, particularly with respect to ADCD and ADNP (Extended Data Fig. 8a).

Next, we determined whether WRAIR NTD and RBD mAb combinations could confer protection in vivo with a series of experiments using the K18-hACE2 transgenic SARS-CoV-2 mouse as described above. To assess protection provided by prophylaxis, potently neutralizing NTD and RBD mAbs were administered either singly or as a 1:1 combination at a low dose of 20 µg (1 mg per kg body weight). K18-hACE2 mice treated with these single or dual mAb combinations did not show any clinical signs of illness during post-challenge follow-up, while weight loss was observed from day 5 in control animals that received the isotype control mAb (ZIKV_MZ4 (ref. ^39^); Fig. 5c). By day 7, animals in the control group succumbed to SARS-CoV-2 infection (Fig. 5c). High infectious virus titer levels were found in lung homogenates, measured at the peak of viral replication, 2 d after infection (Fig. 5c,). While all mAb-treated groups exhibited significantly lower viral titers in the lungs compared to the isotype control group, all animals treated with the mAb combinations demonstrated undetectable virus in the lungs, with the exception of two mice (Fig. 5c). In contrast, low levels of replicating virus were found in mice that received a single mAb at 1 mg per kg body weight (Fig. 5c), supporting the idea that combination of mAbs targeting two different sites on the S protein surface offers enhanced protection.

To determine the minimal protective dose for prophylactic protection for a combination of WRAIR-2039 (NTD) and WRAIR-2123 (RBD), we next titrated the passively administered potent neutralizing mAbs until protection was lost (Fig. 5d). In a 1:1 combination, WRAIR-2139 (NTD) and WRAIR-2123 (RBD) provided suppression of viral replication in the lungs at a low dose of 5 µg (0.25 mg per kg body weight), where each mAb was used at a dose of 2.5 µg or 0.125 mg per kg body weight (Fig. 5d). In addition to prophylaxis, we assessed whether NTD-targeting and RBD-targeting mAb combinations could provide therapeutic benefit, 1 d after challenge in the same K18 mice model. A dose-titration experiment revealed that 50 µg (2.5 mg per kg body weight) of the NTD mAb WRAIR-2039 in combination with RBD mAb WRAIR-2125 was fully protective, with partial protection (4 of 10 animals) observed at a dose of 12.5 µg (0.625 mg per kg body weight; Fig. 5e), demonstrating high potency of mAb combinations in both prophylactic and therapeutic challenge models.

Targeting two different sites on the S protein surface may also prevent the emergence of antibody resistant viral variants. To test this hypothesis, we cultivated rVSV/SARS-CoV-2/GFP in the presence of single NTD and RBD mAbs, and subsequently selected for resistant viral populations that replicated to high levels, as expected (Fig. 5f). In contrast, when dual combinations containing NTD and RBD mAbs were used at the same total concentration (10 μg ml^−1^) as was used for the individual mAbs, no infectious rVSV/SARS-CoV-2/GFP was recovered (Fig. 5f). Thus, consistent with previous observations^36^, S mutations can be readily acquired causing escape from individual antibodies, but mAb combinations that target distinct epitopes present a higher genetic barrier to viral escape. Collectively, NTD and RBD mAb combinations demonstrate complementary antibody functions, enhanced in vivo protection and provide higher resistance to viral escape.

### Coverage of NTD-directed and RBD-directed antibodies across variants of concern

**Fig. 6:**
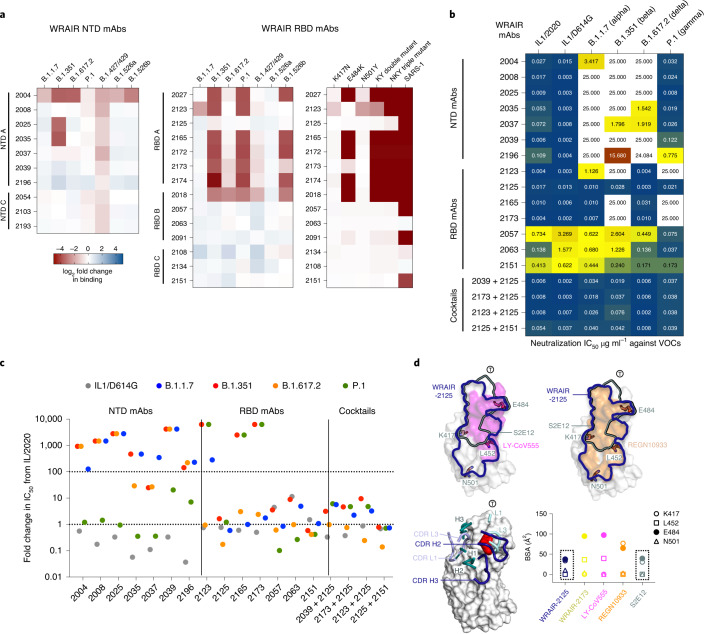
WRAIR mAb binding and neutralization against current circulating variants of concern. **a**, Binding of NTD-directed (left) and RBD-directed (middle) mAbs to stabilized S trimer (S-2P) or RBD mutants (right) harboring mutations present in VOCs and VOIs assessed by BLI. The heat map shows the log_2_ fold change in binding relative to WA1/2020 D614G S-2P spike or WA1/2020 RBD proteins with loss and gain in binding represented in shades of red and blue, respectively. **b**, Neutralization activity of NTD and RBD mAbs, either singly or in combination, against a panel of pSVs representing the current circulating VOCs. The heat map indicates IC_50_ values (µg ml^−1^) ranging from very potent (dark blue), to intermediate (yellow), to poorly neutralizing (dark red), with non-neutralizing mAbs in white. **c**, Same data as in **b** but represented as fold change in IC_50_ relative to the IL1/2020 virus. Neutralization escape is defined as a fold increase in IC_50_ > 100. **d**, Top, comparison of epitopes (outlined) between WRAIR-2125 (blue), S2E12 (green), REGN10933 and LY-CoV555 with RBD neutralization escape residues shown in stick representation. Bottom: left, WRAIR-2125 (blue) and S2E12 (green) heavy-chain and light-chain CDR loop contact residues are shown in ribbon representation, with the RBD shown in surface representation; residue F486 is highlighted in red; right, antibody BSAs for the four RBD residues that differ in VOCs/VOIs. Source data

Finally, the emergence of several viral VOCs threatens current preventative and therapeutic strategies using SARS-CoV-2 neutralizing mAbs. To evaluate the activity of WRAIR mAbs against VOCs, we first assessed binding against a set of S trimers harboring mutations found in circulating VOCs (Alpha, Beta, Delta and Gamma strains) and two variants of interest (VOIs; B.1.427/429 and B.1.526a/b). NTD mAbs showed up to an eightfold reduced binding to B.1.351 (Beta) and 2- to 3-fold reduced binding to B.1.427/429, but most retained binding to B.1.1.7 (Alpha), B.1.617.2 (Delta) and P.1 (Gamma; Fig. 6a). However, even when binding was detected, NTD mAbs exhibited altered binding kinetics to B.1.1.7, B.1.351 and B.1.617.2 S trimers, manifested by slower association (decrease in on-rate) and/or faster dissociation (increase in off-rate) (Extended Data Fig. 8b). RBD mAbs were tested against the same panel of S variants. For RBD A mAbs, loss of binding was largely driven by the p.Glu484Lys mutation, especially when combined with other RBD residue changes such as p.Lys417Asn/Thr and p.Asn501Tyr (found in the B.1.351 (Beta) and P.1 (Gamma) variants; Fig. 6a). Binding to RBD proteins harboring those three mutations, both individually and in combinations, confirmed these results (Fig. 6a). Among potent neutralizing mAbs, RBD mAb WRAIR-2125 retained binding to all VOCs tested, while RBD mAb WRAIR-2173 binding was ablated by the combined double and triple mutations found in VOCs such as B.1.351 and P.1 (Fig. 6a). As expected, binding of RBD mAbs from competition groups B and C were less affected by these mutations as their epitopes lie outside the ACE2 binding interface (Fig. 3). Neutralizing RBD B mAb WRAIR-2063 bound equally well to all wild-type and mutant proteins, including SARS-CoV-1 (Sino 1-11) RBD (Fig. 6a). We next performed pSV neutralization assays against a panel of SARS-CoV-2 strains encompassing the original virus and circulating VOCs. Several mutations such as 69-70del and Tyr144del (B.1.1.7), 241-243del (B.1.351) or 156-157del (B.1.617.2) conferred SARS-CoV-2 resistance to NTD-mediated neutralization (Fig. 6b,c). As a result, most WRAIR NTD neutralizing mAbs lost their activity against pseudotyped B.1.1.7 (Alpha), B.1.351 (Beta) and B.1.617.2 (Delta), but, interestingly, retained intact potency against P.1 (Gamma), indicating that the mutations present in the NTD of this variant are not as disruptive (Fig. 6b,c). However, both WRAIR-2035 and WRAIR-2037 retained modest neutralizing activity against B.1.617.2 (Delta), while the latter also neutralized B.1.351 (Beta). For the WRAIR RBD mAbs, several remained highly potent against the B.1.1.7 (Alpha) variant, which harbors a single RBD mutation, at position p.Asn501Tyr. Similarly, the mutations p.Leu452Arg and p.Thr478Lys present in the B.1.617.2 (Delta) variant did not impact the neutralization activity of the most potent RBD mAbs such as WRAIR-2123 and WRAIR-2125, which both displayed an IC_50_ value of 3-4 ng ml^−1^ against this currently dominating variant. Other variants such as B.1.351 (Beta) and P.1 (Gamma), which combine mutations p.Lys417Asn/Thr, p.Glu484Lys and p.Asn501Tyr, escaped pSV neutralization from most RBD A mAbs, including three of the most potent WRAIR mAbs, WRAIR-2123, WRAIR-2165 and WRAIR-2173. Remarkably, and in agreement with its ability to bind to S trimers harboring mutations found in VOCs, WRAIR-2125 was the only RBD A mAb able to potently neutralize all VOCs (Fig. 6b,c). RBD mAbs targeting epitopes outside the ACE2 binding interface, such as WRAIR-2057, WRAIR-2063 and WRAIR-2151, were also able to neutralize all SARS-CoV-2 strains tested, albeit less potently than WRAIR-2125 (Fig. 6b,c). In addition, antibody combinations comprising WRAIR-2125 and either the NTD mAb WRAIR-2039 or the RBD mAbs WRAIR-2123, WRAIR-2173 or WRAIR-2151 demonstrated potent neutralization across all VOCs (Fig. 6b,c). Taken together, multiple sets of residue mutations and deletions impact antibody binding and neutralization. However, remarkably, WRAIR-2125, retained potent neutralization activities against all VOCs either alone or in combination with NTD or other RBD mAbs.

### Structural basis for WRAIR-2125 exceptional breadth

To further understand WRAIR-2125 antibody recognition of the SARS-CoV-2 RBD, we juxtaposed its contact residues in comparison to other known mAbs. In contrast to other RBD class 1 mAbs, WRAIR-2125 does not interact with residue N501 and has reduced BSA interaction with both E484 and K417 residues, explaining its ability to resist neutralization escape by VOCs (Fig. 6d).

Comparing the antibody gene usage and targeted epitope to previously published antibodies (Supplementary Table 1), WRAIR-2125 shares heavy-chain (IGHV3-30*18) and light-chain (IGKV1–39*01) genes with recurring antibodies observed in multiple donors^14^, but with alternate D (IGHD3–22) and J (IGHJ1) gene combinations specific to WRAIR-2125. The WRAIR-2125 epitope shares a resemblance to a class of F486-targeting IGHV1–58/IGKV3–20-derived mAbs such as S2E12 that belong to a public clonotype^4,16,40^, despite little sequence identity (Supplementary Table 1). In addition, WRAIR-2125 approaches the RBD from a different angle, and uses predominantly CDR H loops to recognize F486 (Extended Data Fig. 5b) contrasting with the recognition mechanism of the public clonotype family. Comparison of WRAIR-2125 and S2E12 epitopes^16^ highlights the overlapping epitope targeted by both mAbs (Fig. 6d). WRAIR-2125 and S2E12 antibody epitopes are largely located on one side of the ACE2 binding site, thereby weakly contacting residues K417 (BSA is 33.7 Å^2^ for 2125 and 30.3 Å^2^ for S2E12) and E484 (BSA 37.4 Å^2^ for 2125 and 39.4 Å^2^ for S2E12; Fig. 6d). Therefore, WRAIR-2125 is a new example of a F486-targeting antibody with broad neutralization potency across all VOCs. Contrasting with the ACE2 binding site recognition of WRAIR-2173, and also first-generation emergency use authorization mAbs REGN10933 and LY-CoV555 (refs. ^1,40,41^), all of which interact with residues frequently mutated in VOCs/VOIs (Fig. 6d). Analysis of the REGN10933 epitope highlights the antibody interactions with residues K417 and E484 burying a total surface area of 77.0 Å^2^ and 64.8 Å^2^, respectively, and additional interactions with residue N501. Antibody LY-COV555, forms large contacts with residue E484 with a total BSA of 97.3 Å^2^ (Fig. 6d). Similarly, WRAIR-2173 also forms a strong contact with residue E484 (BSA 94.7 Å^2^) and minor contacts with N501 (BSA 3.6 Å^2^), resulting in a lack of neutralization with B.1.351 (Beta) while still maintaining robust neutralization of other VOCs. Variations in antibody recognition highlight the advantages of optimal antibody combinations that can be exploited for next-generation therapeutic use, with WRAIR-2125 having an advantage against current circulating VOCs.

## Discussion

In this study, we isolated potent neutralizing mAbs targeting the NTD supersite and RBD on the surface of the viral S glycoprotein, adding to the current arsenal of potent neutralizing antibodies described^1,3,7,8,12–15,17,20,22,27,29,37,40–45^. NTD-targeting neutralizing antibodies likely impede the normal SARS-CoV-2 S function by interfering with the fusion of virus and host cell membranes via steric hindrance^7,8,22,46^, or as previously reported for MERS-CoV NTD-targeting and neutralizing antibody 7D10, by preventing protease cleavage of S^38^. In addition to neutralization, both NTD-targeting and RBD-targeting mAbs were capable of mediating Fc effector functions, with a unique ability of NTD neutralizing mAbs to leverage complement deposition. Our data suggest that the NTD mAbs bind with higher affinity to the S glycoprotein than the RBD-targeting mAbs, which may offer benefits in mediating Fc effector functions. As these NTD and RBD mAbs do not compete for binding to the S trimer, several combinations of NTD and RBD mAbs were tested for neutralizing capacity and in vivo protection. Combinations of NTD and RBD mAbs demonstrated complementary effects on viral neutralization and Fc effector functions in vitro and yielded potent in vivo prophylactic and therapeutic efficacy. When administered prophylactically, complete protection was observed at a low dose of 20 µg (1 mg per kg body weight), and partial protection at a dose of 5 µg (0.25 mg per kg body weight), while therapeutic efficacy was observed at 2.5 mg per kg body weight. Prophylactic in vivo protection by NTD-targeting, but not RBD-targeting mAbs, required an intact IgG1 Fc domain, underlining the importance of Fc effector functions for NTD-targeting mAbs in mediating protection. Along with ADNP, engagement of complement (ADCD) has been associated with survival from COVID-19 (ref. ^47^), and synergy between Fab and Fc effector functions has been shown to be critical for vaccine-elicited protection^48^.

VOCs B.1.1.7, B.1.351, P.1 and B.1.617.2, harboring multiple mutations in both NTD and RBD domains, have been shown to escape first-generation mAb therapeutics^9,10^. As such, there is a need for prophylactic and therapeutic mAbs with broad and potent activity against all circulating SARS-CoV-2 strains. Remarkably, WRAIR-2125 potently neutralized all VOCs ranging from 3 to 28 ng ml^−1^. WRAIR-2125 was found to target a minimal epitope required for ACE2 engagement on the RBD, centered around residue F486, with minimal interactions with residues mutated in VOCs such as E484, found to be important for binding of other potent neutralizing RBD mAbs.

Consistent with previous studies, the majority of isolated SARS-CoV-2 mAbs were similar to germline sequences, in agreement with the observation that germline-encoded residues play an important role in the binding of several potent neutralizing antibodies across multiple classes^4,49^. Altogether, these data demonstrate that NTD-RBD mAb combinations offer low-dose protection in vivo, likely leveraging complementary Fab-mediated and Fc-mediated antiviral activities. Combinations prevented viral escape and provided stronger coverage across current circulating VOCs. These data indicate that mAb combinations offer advantages to combat SARS-CoV-2 current and future variants, especially in immunocompromised populations or individuals who do not respond to vaccination.

## Methods

### Human samples

All authors have complied with the ethical regulations regarding these studies. These studies were approved by the WRAIR Institutional Review Board, and written informed consent was obtained from all participants. The investigators have adhered to the policies for protection of human participants as prescribed in AR 70–25. Plasma from healthy and SARS-CoV-2 convalescent donors originated from WRAIR RV229 and RV229H studies, respectively. Other sources for convalescent plasma included StemExpress and the National Institute for Allergy and Infectious Diseases (NIAID) through its Biodefense and Emerging Infections research (BEI) repository. All convalescent donors (44% male and 56% female, aged between 30 and 65 years) experienced a range of mild to severe symptoms, with blood drawn 3–7 weeks following the onset of symptoms. Donor 3, from whom mAbs were isolated, was enrolled in the RV229H study after experiencing mild to moderate symptoms. Plasma and PBMCs were collected 7 weeks following a SARS-CoV-2-positive PCR test.

### Multiplex antibody binding assay

A high-throughput bead-based antibody binding assay was performed as previously described^50,51^ with modifications to adapt to coronavirus antigens. A cocktail of 25 coronavirus antigens and 2 control proteins (HIV-1 antigens), obtained commercially (Sino Biological) or internally produced (see below), spanning spike S1 and S2 domains for all seven human coronaviruses were covalently coupled to uniquely coded magnetic microspheres (Luminex) per the manufacturer’s protocol. Data were collected on a Bio-Plex 3D Suspension Array system (Bio-Rad) running xPONENT v.4.2 (Luminex). Signal-to-noise ratios were calculated by the dividing the MFI for each sample by either immunoglobulin-depleted healthy plasma or a negative control antibody (MZ4) according to the type of sample analyzed.

### SARS-CoV-2 pseudovirus neutralization assay

SARS-CoV-2 pseudovirions (pSV) were produced by co-transfection of HEK293T/17 cells with a pcDNA3.1 encoding SARS-CoV-2 S protein and an HIV-1 NL4-3 luciferase reporter plasmid (pNL4-3.Luc.R-E-, National Institutes of Health (NIH) AIDS Reagent Program). The S expression plasmid sequence was derived from the Wuhan Hu-1 strain (GenBank, NC_045512), which is also identical to the IL1/2020 and WA1/2020 strains. The S expression plasmid sequence was also codon optimized and modified to remove the last 18 amino acids of the cytoplasmic tail to improve S incorporation into the pseudovirions and thereby enhance infectivity. S expression plasmids for current SARS-CoV-2 VOCs and VOIs were similarly codon optimized, modified and included the following mutations: B.1.1.7 or Alpha, (69-70del, Tyr144del, p.Asn501Tyr, p.Ala570Asp, p.Asp614Gly, p.Pro681His, p.Thr718Ile, p.Ser982Ala and p.Asp1118His), B.1.351 or Beta, (p.Leu18Phe, p.Asp80Ala, p.Asp215Gly, 241-243del, p.Lys417Asn, p.Glu484Lys, p.Asn501Tyr, p.Asp614Gly, p.Ala701Val and p.Glu1195Gln), B.1.617.2 or Delta, (p.Thr19Arg, p.Gly142Asp, del156-157, p.Arg158Gly, p.Leu452Arg, p.Thr478Lys, p.Asp614Gly, p.Pro681Arg and p.Asp950Asn), P.1 or Gamma (p.Leu18Phe, p.Thr20Asn, p.Pro26Ser, p.Asp138Tyr, p.Arg190Ser, p.Lys417Thr, p.Glu484Lys, p.Asn501Tyr, p.Asp614Gly, p.His655Tyr and p.Thr1027Ile) and B.1.427/429 (p.Ser13Ile, p.Trp152Cys, p.Leu452R and p.Asp614Gly). An Asp614Gly variant was also made from the Wuhan Hu-1 construct using the Q5 site-directed mutagenesis kit (NEB). In addition, a codon-optimized S expression plasmid encoding SARS-CoV-1 (Sino 1-11; GenBank, AY485277) was generated that incorporated a 28 amino acid C-terminal deletion to improve infectivity^52^. Virions pseudotyped with the VSV G protein were used as control. Infectivity and neutralization titers were determined using ACE2-expressing HEK293 target cells (Integral Molecular) in a semiautomated assay format using robotic liquid handling (Biomek NXp Beckman Coulter), as previously described^18^. Neutralization dose–response curves were fitted by nonlinear regression using the LabKey server, and the final titers are reported as the reciprocal of the dilution of plasma necessary to achieve 50% neutralization (50% inhibitory dose (ID_50_) or IC_50_) and 80% neutralization (80% inhibitory dose (ID_80_) or 80% inhibitory concentration (IC_80_)). Assay equivalency was verified by participation in the SARS-CoV-2 Neutralizing Assay Concordance Survey run by the Virology Quality Assurance Program and External Quality Assurance Program Oversite Laboratory at the Duke Human Vaccine Institute, sponsored through programs supported by the NIAID, Division of AIDS.

### Sorting of SARS-CoV-2-positive B cells

Cryopreserved PBMCs were thawed in warm medium containing benzonase, then washed with PBS and stained for viability using the Aqua Live/Dead stain (Thermo Fisher). Cells were incubated at 21 °C for 30 min with a cocktail of antibodies including CD3 BV510 (BioLegend), CD4 BV510 (BD Biosciences), CD8 BV510 (BioLegend), CD14 BV510 (BioLegend), CD16 BV510 (BD Biosciences) and CD56 BV510 (BioLegend) as dump channel markers, and CD19 PE Dazzle 594 (BioLegend), CD38 BUV496 (BD Biosciences), CD27 BV605 (BioLegend), CD20 AF700 (BD Biosciences), IgD APC/Cyanine7 (BioLegend), integrin β7 PE/Cyanine7 (BD Biosciences), IgG (BioLegend), CD10 BUV395 (BD Biosciences), CD21 FITC (BioLegend) and IgM BV650 (BioLegend). Two sorting strategies were used to maximize the number of probes used to isolate antigen-specific B cells: the first strategy utilized a stabilized SARS-CoV-2 S trimer (HexaPro^11^) conjugated to streptavidin-APC, and the second strategy utilized a multivalent SpFN^18^ displaying eight S trimers to potentially capture conformation-specific B cell receptors. SpFN was incubated with cells during primary staining, and SpFN^+^ B cell were identified by secondary staining using the MM43 mAb (Sino Biological, 40591-MM43) conjugated to AF647 (Thermo Fisher). Both strategies included SARS-CoV-2 RBD, S1 and S2 (Thermo Fisher), which were biotinylated, tetramerized and conjugated to streptavidin-PE. Because these antigens used the same conjugated streptavidin-PE, B cell binding could not be distinguished between SARS-CoV-2 RBD, S1 and S2 using flow cytometry. Specific B cell binding by flow cytometry to the stabilized trimer was determined using conjugated APC, and SpFN using AF647 conjugated to MM43. CD19^+^ B cells that were antigen specific were single-cell sorted into PCR plates containing lysis buffer composed of murine RNase inhibitor (New England Biolabs), dithiothreitol, SuperScript III First Strand Buffer (Thermo Fisher), Igepal (Sigma) and carrier RNA (Qiagen) at one cell per well using a FACSAria (Becton Dickinson) and stored at −80 °C until subsequent reverse transcription. Analysis was performed using FlowJo v10 (BD Bioscience).

### Antibody sequencing and production

RNA from single antigen-specific B cells was reverse transcribed using random hexamers and the SuperScript III kit (Thermo Fisher). Antibody V(D)J genes were amplified from the cDNA by nested PCR, with the HotStar Taq DNA Polymerase kit (Qiagen) using a combination of primer sets and methods described previously^39^. V(D)J gene assignment, somatic hypermutation and CDR3 determinations were performed using IgBLAST. Antibody variable regions were synthesized and cloned (GenScript) into CMVR expression vectors (NIH AIDS reagent program) between a mouse immunoglobulin leader (GenBank, DQ407610) and the constant regions of human IgG1 (GenBank, AAA02914), Igκ (GenBank, AKL91145) or Igλ (GenBank, AAA02915). Antibodies were expressed by co-transfecting plasmids encoding paired heavy and light chains into Expi293F cells (Thermo Fisher). Monoclonal antibodies were purified 4 to 5 d after transfection using AmMag protein-A magnetic beads and the AmMag SA purification system (GenScript), according to the manufacturer’s recommendations, and buffer exchanged into PBS. The purity and stability of mAbs was assessed by SDS–PAGE and Coomassie staining in both reducing and non-reducing conditions. Control antibodies were all expressed as human IgG1 and purified from Expi293F cells, as described above.

### Fab production

Freshly purified WRAIR IgGs in PBS buffer (pH 7.4) were mixed with Lys C protease (New England Biolabs) at a 1:2,000 (wt:wt) ratio. The reaction was allowed to proceed for 2–3 h in a water bath incubator at 37 °C. Digestion was assessed by SDS–PAGE and, upon completion, the reaction mixture was passed through protein-A beads (0.5–1-ml beads) three times and the final flow through was assessed by SDS–PAGE for purity.

### Production of recombinant proteins

Recombinant SARS-CoV-2 proteins RBD (318–514), NTD (1–290) and S1 (1–665) were made from a synthesized full-length spike sequence (GenScript) from strain USA/IL1/2020 (GenBank, MN988713) and were cloned with C-terminal AviTag and poly-histidine tags into the CMVR vector under the bovine prolactin leader sequence. The coding sequence for the SARS-CoV-2 (GenBank, MN908947) stabilized trimer (S-2P) was a generous gift from J. McLellan. The S-2P sequence was subcloned into the pCMVR vector with C-terminal AviTag and poly-histidine tags. Four additional stabilizing mutations were added using the QuikChange multisite-directed mutagenesis kit (Agilent) to make the HexaPro variant with improved stability^11^, referred to as stabilized S trimer throughout the paper. SARS-CoV-2 RBD constructs (331–527), also modified to incorporate an N-terminal hexa-histidine tag, were derived from the Wuhan Hu-1 strain genome sequence (GenBank, MN908947.3). Subsequent RBD VOCs with point mutations were generated using a modified QuikChange site-directed mutagenesis protocol (Agilent). An S-2P construct derived from SARS-CoV-1 was generated as previously described^53^. Spike proteins were expressed and biotinylated as previously described^54^, with mutations for B.1.1.7, B.1.351, P.1, B.1.617.2 and other variants added by QuikChange site-directed mutagenesis. ACE2-Ig, a fusion protein made by connecting the human ACE2 (Q9BYF1) extracellular domain (residues 19–611) to the constant domain of a human IgG1 was expressed and purified as described above for antibodies. All proteins were produced transiently from Expi293F or FreeStyle 293F (stabilized trimer) cells (both Thermo Fisher) and purified from cell culture supernatants using Ni-NTA (Qiagen) affinity. The stabilized trimer was further purified by gel filtration on an ENrich SEC 650 column (Bio-Rad) and the presence of trimeric S was verified by negative-stain EM analysis. When needed, proteins were biotinylated using the BirA biotin-protein ligase kit (Avidity).

### Authentic SARS-CoV-2 plaque reduction neutralization test

Vero E6 cells (American Type Culture Collection CRL-1586) maintained in DMEM medium supplemented with 10% FBS and 2 mM l-glutamine were seeded in six-well plates at 1 × 10^6^ cells per well 1 d before infection. Plaque reduction neutralization tests (PRNTs) were performed in triplicate in a biosafety level 3 facility. Threefold dilutions were performed for each mAb, beginning at 25 µg ml^−1^. The dilutions were made at 2× concentrations and mixed at a 1:1 ratio with 100 PFUs of SARS-CoV-2 virus (isolate 2019-nCoV/Italy-INMI1, BEI NR-52284, which is 100% identical to the Wuhan Hu-1 or IL1/2020 strains). The antibody–virus mixtures were incubated at 37 °C for 1 h. The mixtures were then added to the Vero E6 monolayers, incubated for 1 h at 37 °C in a humidified incubator with 5% CO_2_, then overlaid with 0.5% agarose in serum-free minimal essential media with 100 U ml^−1^ of penicillin–streptomycin, 0.25 µg ml^−1^ amphotericin B and 2 mM l-glutamine. The cells were incubated for 72 h, then fixed in 10% formaldehyde and stained with 0.5% crystal violet. The IC_50_ values were determined as the concentration of antibody that resulted in a 50% reduction in number of plaques, compared to virus-only control wells.

### Measurements of antibody Fc effector functions using recombinant proteins

#### Antibody-dependent cellular phagocytosis

ADCP was measured as previously described^55^ using biotinylated SARS-CoV-2 S stabilized trimer. The phagocytic score was calculated by multiplying the percentage of bead-positive cells by the geometric MFI of the bead-positive cells and dividing by 10^4^.

#### Antibody-dependent neutrophil phagocytosis

Biotinylated SARS-CoV-2 stabilized trimer was incubated with yellow-green streptavidin fluorescence beads (Molecular Probes) for 2 h at 37 °C. Next, 10 μl of a 100-fold dilution of beads–protein mixture was incubated with mAbs as described above before addition of effector cells (50,000 cells per well). Fresh peripheral blood leukocytes from human samples were used as effector cells after red blood cell lysis with ACK lysing buffer (Thermo Fisher Scientific). After 1 h of incubation at 37 °C, the cells were washed, surface stained, fixed with 4% formaldehyde solution and fluorescence was evaluated on an LSR II (BD Bioscience). Antibodies used for flow cytometry were anti-human CD3 AF700 (clone UCHT1) and anti-human CD14 APC-Cy7 (clone MϕP9; both BD Biosciences) and anti-human CD66b Pacific Blue (clone G10F5, BioLegend). The phagocytic score was calculated by multiplying the percentage of bead-positive neutrophils (SSC^hi^CD3^−^CD14^−^CD66^+^) by the geometric MFI of the bead-positive cells and dividing by 10^4^.

### Measurements of antibody Fc effector functions using cell-surface-expressed spike proteins

#### Opsonization

SARS-CoV-2 S-expressing FreeStyle 293F cells were generated by transfection with linearized plasmid encoding a codon-optimized full-length SARS-CoV-2 S protein matching the amino acid sequence of the IL1/2020 isolate (GenBank, MN988713). Stable transfectants were single-cell sorted and selected to obtain a high-level spike surface expressing clone (293F-Spike-S2A). 293F-Spike-S2A cells were incubated with mAbs diluted threefold from 15 to 0.06 µg ml^−1^ for 30 min at 37 °C. Cells were washed twice and stained with anti-human IgG PE (Southern Biotech). Cells were then fixed with 4% formaldehyde solution and fluorescence was evaluated on an LSR II (BD Bioscience).

#### Antibody-dependent complement activation

An ADCD assay was adapted from work by Fischinger et al.^56^. Briefly, 293F-Spike-S2A cells were incubated with mAbs as described above and washed twice and resuspended in R10 media. Cells were washed with PBS and resuspended in 200 µl of guinea pig complement (Cedarlane), which was prepared at a 1:50 dilution in Gelatin Veronal Buffer with Ca^2+^ and Mg^2+^ (Boston BioProducts). After incubation at 37 °C for 20 min, cells were washed and stained with an anti-guinea pig complement C3-FITC (polyclonal, Thermo Fisher Scientific). Cells were fixed with 4% formaldehyde solution and fluorescence was evaluated on an LSR II (BD Bioscience).

### Epitope binning

Epitopes of the NTD and RBD mAbs were first mapped by binding competition against each other (NTD) or against a set of control antibodies (RBD) using BLI on an Octet RED96 instrument (FortéBio), as previously described^39^. Antibodies were defined as competing when binding signal of the second antibody was reduced to less than 25% of its maximum binding capacity and non-competing when binding was greater than 50%. Intermediate competition was defined by binding levels of 25–50%. Control antibodies RBD A, RBD B and RBD C were CC12.1, CC12.16 (ref. ^13^) and CR3022 (ref. ^57^), respectively. The same approach was used to assess binding competition between NTD and RBD antibodies within the stabilized S trimer. ACE2-Ig was used like an antibody to assess the ability of NTD and RBD antibodies to block ACE2 binding to the S trimer.

### Biolayer interferometry binding assays

Real-time interactions between purified SARS-CoV-2 proteins and antibodies were monitored on an Octet RED96 instrument (FortéBio) as previously described^39^ using biotinylated SARS-CoV-2 NTD and RBD proteins as described above. After reference subtraction, apparent binding kinetic constants were determined, from at least four concentrations of antibody, by fitting the curves to a 1:1 binding model using the data analysis software 10.0 (FortéBio). To assess binding to a panel of RBD mutants, HIS1K biosensors (FortéBio) were equilibrated in assay buffer (PBS) for 15 s before loading of His-tagged SARS-CoV-2 RBD, VOC RBDs or SARS-CoV-1 RBD (30 μg ml^−^^1^ diluted in PBS) for 100 s. Binding responses were measured at the end of the association step using the data analysis software 10.0 (FortéBio). ACE2-RBD competition assays were carried out as follows: SARS-CoV-2 RBD (30 μg ml^−1^ diluted in PBS) was immobilized on HIS1K biosensors (FortéBio) for 220 s. Test antibodies were allowed to bind for 200 s, followed by baseline equilibration (30 s), and then incubation with ACE2 protein (30 μg ml^−1^) for 120 s. Percentage inhibition (PI) of RBD binding to ACE2 by antibodies was determined using the equation: PI = ((ACE2 binding following RBD-antibody incubation)) ⁄ (ACE2 binding)) × 100. Antibody concentration was titrated from 100 μg ml^−1^ by serial twofold dilutions. All assays were performed at 30 °C with agitation set at 1,000 r.p.m.

### Epitope mapping of antibodies by alanine scanning

Epitope mapping was performed essentially as described previously^58^ using SARS-CoV-2 (strain Wuhan Hu-1) S protein RBD and NTD shotgun mutagenesis mutation libraries, made using a full-length expression construct for S protein. In total, 184 residues of the RBD (between S residues 335 and 526), and 300 residues of the NTD (between residues 2 and 307) were mutated individually to alanine, and alanine residues to serine. Mutations were confirmed by DNA sequencing, and clones arrayed in 384-well plates, with one mutant per well. Binding of mAbs to each mutant clone in the alanine scanning library was determined, in duplicate, by high-throughput flow cytometry. Antibody reactivity against each mutant S protein clone was calculated relative to wild-type S protein reactivity by subtracting the signal from mock-transfected controls and normalizing to the signal from wild-type S-transfected controls. Mutations within clones were identified as critical to the mAb epitope if they did not support reactivity of the test mAb but supported reactivity of other SARS-CoV-2 antibodies. This counter-screen strategy facilitates the exclusion of S mutants that are locally misfolded or have an expression defect.

### X-ray crystallography and structure analysis

WRAIR-2173–RBD (15.0 mg ml^−1^), WRAIR-2151–RBD (12.0 mg ml^−1^), WRAIR-2057–RBD (12.0 mg ml^−1^) and WRAIR-2125–RBD complexes (10.0 mg ml^−1^) were screened for crystallization conditions using an Art Robbins Gryphon crystallization robot, 0.2-µl drops, and a set of 1,200 conditions and observed daily using a Jan Scientific UVEX-PS. Crystals used for data collection grew in the following crystallization conditions: WRAIR-2173–RBD complex: 0.09 M NPS (sodium nitrate, sodium phosphate dibasic and ammonium sulfate), 0.1 M buffer system 3 (Tris base and BICINE, pH 8.5), 50% precipitant mix 4 (25% vol/vol MPD; 25% PEG 1000; 25% wt/vol PEG 3350); WRAIR-2151–RBD complex: 0.1 M sodium acetate trihydrate (pH 4.6), 2.0 M ammonium sulfate; WRAIR-2057–RBD complex: 8% vol/vol Tacsimate (pH 5.0), 20% wt/vol polyethylene glycol 3,350; WRAIR-2125–RBD complex: 0.12 M alcohol mixture (1,6-hexanediol, 1-butanol, 1,2-propanediol, 2-propanol, 1,4-butanediol and 1,3-propanediol), 0.1 M buffer system 3 (Tris base and bicine, pH 8.5), 50% precipitant mix 4 (25% vol/vol MPD, 25% PEG 1000 and 25% wt/vol PEG 3350) and 0.1 M manganese(II) chloride tetrahydrate.

Diffraction data were collected at Advanced Photon Source beamlines. Diffraction data for WRAIR-2125–RBD and WRAIR-2151–RBD complexes were notably anisotropic and were corrected using the UCLA Diffraction Anisotropy Server^59^. All the crystal structures described in this study were solved by molecular replacement using PHASER, and iterative model building and refinement were performed in COOT and Phenix^60–62^. Diffraction data quality was assessed with Phenix xtriage using data output from HKL2000 (ref. ^63^) and XDS. Data collection, molecular replacement search models and refinement statistics are reported in Supplementary Table 2. All structures were refined using Phenix refine with positional, global isotropic B-factor refinement and defined TLS groups. Manual model building was performed in COOT. Overall, the Ramachandran plot as determined by MOLPROBITY showed 92–95% of all residues in favored regions and 4–6% of all residues in the allowed regions. Electron density for the structures was clearly interpretable except for the heavy-chain Fc1 domain of WRAIR-2151. Interactive surfaces were analyzed using PISA (https://www.ebi.ac.uk/pdbe/pisa/; Supplementary Table 3). Structure figures were prepared using PyMOL (DeLano Scientific).

### Negative-stain electron microscopy

Fab fragments and SARS-CoV-2 S-2P were mixed at a 3:1 molar ratio for 30 min at room temperature, followed by purification using a Superdex-200 column. Purified proteins (5–10 μg ml^−1^) were deposited on carbon-coated copper grids and stained with 0.75% uranyl formate and imaged using a FEI T20 operating at 200 kV with an Eagle 4 K CCD using SerialEM or using a Thermo Scientific Talos L120C operating at 120 kV with Thermo Scientific Ceta detector using EPU. All image processing steps were done using RELION (v3.0.8)^64^ and cryosparc (v3.2.0)^65^. Particles were picked either manually or using templates generated from manually picked two-dimensional class averages. Contrast transfer function estimation was performed with CTFFIND 4.1.13 and used for two-dimensional classification. Three-dimensional map reconstructions were generated using an initial reference generated from S-2P (Protein Data Bank (PDB) 6VXX) with a low-pass filter of 100 Å to remove distinguishable features and ‘C1’ symmetry. An intermediate structure model was used to create a mask to further refine the structure. Visual analysis and figure generation were performed using Chimera^66^.

### In vivo protection studies in K18-hACE2 transgenic mice

All research in this study involving animals was conducted in compliance with the Animal Welfare Act, and other federal statutes and regulations relating to animals and experiments involving animals and adhered to the principles stated in the Guide for the Care and Use of Laboratory Animals, NRC Publication, 1996 edition. The research protocol was approved by the Institutional Animal Care and Use Committee of the Trudeau Institute and US Army Medical Research. K18-hACE2 transgenic mice were obtained from Jackson Laboratories. Mice were housed in the animal facility of the Trudeau Institute and cared for in accordance with local, state, federal and institutional policies in an NIH American Association for Accreditation of Laboratory Animal Care-accredited facility. For the prophylactic protection studies, on day −1, groups of 15 male and female K18-hACE2 mice (8–10 weeks of age) were injected intravenously with the purified antibodies at the indicated dose. On study day 0, all mice were inoculated with 1.25 × 10^4^ PFUs of SARS-CoV-2 USA-WA1/2020 via intranasal instillation, a challenge dose determined from a previous study^19^. In the therapeutic study, mice (8–10 weeks of age) were inoculated with SARS-CoV-2 USA-WA1/2020 24 h before being injected intravenously with the indicated antibody cocktail. All mice were monitored for clinical symptoms and body weight twice daily, every 12 h, from study day 0 to study day 14. Mice were euthanized if they displayed any signs of pain or distress as indicated by the failure to move after stimulated or inappetence, or if mice had greater than 25% weight loss compared to their study day 0 body weight. From each group, a subset (five) of mice, were killed 2 d after challenge for determination of infectious virus titers in the lower respiratory tract (from bronchoalveolar lavage and lung tissue) using a PRNT assay.

### Evaluation of escape and selection of virus variants

For the evaluation of antibody escape ability, and generation of putative antibody escape S variants, a previously described chimeric recombinant VSV derivative (rVSV/SARS-CoV-2/GFP2E1) that encodes a SARS-CoV-2 S protein in place of VSV-G, recapitulating the neutralization properties of authentic SARS-CoV-2, was prepared and passaged to generate diversity as previously described^67^.

### Statistical analysis

Neutralization is the geometric mean of the IC_50_ values calculated using five-parameter logistic regression from at least two independent experiments performed in triplicates (R package nplr). Non-parametric Spearman correlations were used to assess relationship between neutralization and binding or neutralization and effector function data as well as between neutralization data obtained from the pseudotyped and authentic SARS-CoV-2 neutralization assays. Two-tailed Mann–Whitney *t*-tests were used to verify the existence of significant differences between NTD and RBD mAbs in several binding and functional assays. In the animal studies, one-way ANOVA with Dunnett’s multiple-comparisons tests were used to assess significance in weight changes and viral loads across groups compared to the isotype control antibody-treated animals. Survival curves were compared individually to the isotype control antibody using a Mantel–Cox log-rank test. Fold change in binding to mutant proteins was calculated relative to the wild-type WA1/2020 S or RBD proteins. In the absence of binding, a background binding value (0.05 nm in BLI assays) was attributed. Fold change in neutralization to VOCs was calculated relative to the IL1/2020 virus. Non-neutralizing mAbs were assigned the IC_50_ value of 25 µg ml^−1^ antibody, the mAb starting concentration in the assay. All tests, except for the five-parameter logistic regression performed in R (version 3.6.3) and R studio (1.2.1355), were performed in Prism (version 9, GraphPad). Data were graphed using Prism (version 9, GraphPad).

### Reporting Summary

Further information on research design is available in the Nature Research Reporting Summary linked to this article.

## Online content

Any methods, additional references, Nature Research reporting summaries, source data, extended data, supplementary information, acknowledgements, peer review information; details of author contributions and competing interests; and statements of data and code availability are available at 10.1038/s41590-021-01068-z.

## Supplementary information

Supplementary Information Supplementary Tables 1 and 2

Reporting Summary

Peer Review Information

Supplementary Table 3 Crystallographic data of mAbs.

## Data Availability

The associated data for the crystallographic complexes reported in this paper are available from the PDB under accession codes 7N4L, 7N4J, 7N4I and 7N4M. The antibody sequences are available at GenBank under accession numbers MZ825470–MZ825529. Source data are provided with this paper. All other data are available from the corresponding authors upon request.

## Source data

Source Data Fig. 1 Statistical source data.
Source Data Fig. 2 Statistical source data.
Source Data Fig. 4 Statistical source data.
Source Data Fig. 5 Statistical source data.
Source Data Fig. 6 Statistical source data.
Source Data Extended Data Fig. 1 Statistical source data.
Source Data Extended Data Fig. 2 Statistical source data.
Source Data Extended Data Fig. 3 Statistical source data.
Source Data Extended Data Fig. 4 Statistical source data.
Source Data Extended Data Fig. 5 Statistical source data.
Source Data Extended Data Fig. 6 Statistical source data.
Source Data Extended Data Fig. 7 Statistical source data.
Source Data Extended Data Fig. 8 Statistical source data.

## References

[1] Gottlieb RL (2021). Effect of bamlanivimab as monotherapy or in combination with etesevimab on viral load in patients with mild to moderate COVID-19: a randomized clinical trial. JAMA.

[2] O’Brien MP (2021). Subcutaneous REGEN-COV antibody combination to prevent COVID-19. N. Engl. J. Med..

[3] Starr TN (2021). SARS-CoV-2 RBD antibodies that maximize breadth and resistance to escape. Nature.

[4] Dong J (2021). Genetic and structural basis for SARS-CoV-2 variant neutralization by a two-antibody cocktail. Nat. Microbiol..

[5] Martinez DR (2021). Prevention and therapy of SARS-CoV-2 and the B.1.351 variant in mice. Cell Rep..

[6] Hoffmann M (2020). SARS-CoV-2 cell entry depends on ACE2 and TMPRSS2 and is blocked by a clinically proven protease inhibitor. Cell.

[7] McCallum M (2021). N-terminal domain antigenic mapping reveals a site of vulnerability for SARS-CoV-2. Cell.

[8] Cerutti G (2021). Potent SARS-CoV-2 neutralizing antibodies directed against spike N-terminal domain target a single supersite. Cell Host Microbe.

[9] Chen RE (2021). Resistance of SARS-CoV-2 variants to neutralization by monoclonal and serum-derived polyclonal antibodies. Nat. Med..

[10] Planas D (2021). Reduced sensitivity of SARS-CoV-2 variant Delta to antibody neutralization. Nature.

[11] Hsieh CL (2020). Structure-based design of prefusion-stabilized SARS-CoV-2 spikes. Science.

[12] Ju B (2020). Human neutralizing antibodies elicited by SARS-CoV-2 infection. Nature.

[13] Rogers TF (2020). Isolation of potent SARS-CoV-2 neutralizing antibodies and protection from disease in a small animal model. Science.

[14] Robbiani DF (2020). Convergent antibody responses to SARS-CoV-2 in convalescent individuals. Nature.

[15] Zost SJ (2020). Potently neutralizing and protective human antibodies against SARS-CoV-2. Nature.

[16] Tortorici MA (2020). Ultrapotent human antibodies protect against SARS-CoV-2 challenge via multiple mechanisms. Science.

[17] Cao Y (2020). Potent neutralizing antibodies against SARS-CoV-2 identified by high-throughput single-cell sequencing of convalescent patients’ B cells. Cell.

[18] Joyce, M. G. et al. Efficacy of a broadly neutralizing SARS-CoV-2 ferritin nanoparticle vaccine in nonhuman primates. Preprint at *bioRxiv*10.1101/2021.03.24.436523 (2021).

[19] Joyce, M. G. et al. SARS-CoV-2 ferritin nanoparticle vaccines elicit broad SARS coronavirus immunogenicity. Preprint at *bioRxiv*10.1101/2021.05.09.443331 (2021). 10.1016/j.celrep.2021.110143 PMC8651551 34919799

[20] Liu L (2020). Potent neutralizing antibodies against multiple epitopes on SARS-CoV-2 spike. Nature.

[21] Sholukh AM (2021). Evaluation of cell-based and surrogate SARS-CoV-2 neutralization assays. J. Clin. Microbiol..

[22] Suryadevara N (2021). Neutralizing and protective human monoclonal antibodies recognizing the N-terminal domain of the SARS-CoV-2 spike protein. Cell.

[23] Winkler ES (2021). Human neutralizing antibodies against SARS-CoV-2 require intact Fc effector functions for optimal therapeutic protection. Cell.

[24] Schafer A (2021). Antibody potency, effector function, and combinations in protection and therapy for SARS-CoV-2 infection in vivo. J. Exp. Med..

[25] Ullah I (2021). Live imaging of SARS-CoV-2 infection in mice reveals that neutralizing antibodies require Fc function for optimal efficacy. Immunity.

[26] Chi X (2020). A neutralizing human antibody binds to the N-terminal domain of the spike protein of SARS-CoV-2. Science.

[27] Voss WN (2021). Prevalent, protective, and convergent IgG recognition of SARS-CoV-2 non-RBD spike epitopes. Science.

[28] Joyce, M. G. et al. A cryptic site of vulnerability on the receptor-binding domain of the SARS-CoV-2 spike glycoprotein. Preprint at *bioRxiv*10.1101/2020.03.15.992883 (2020).

[29] Barnes CO (2020). SARS-CoV-2 neutralizing antibody structures inform therapeutic strategies. Nature.

[30] Dejnirattisai W (2021). The antigenic anatomy of SARS-CoV-2 receptor binding domain. Cell.

[31] Liu H (2021). A combination of cross-neutralizing antibodies synergizes to prevent SARS-CoV-2 and SARS-CoV pseudovirus infection. Cell Host Microbe.

[32] Yuan M (2020). A highly conserved cryptic epitope in the receptor binding domains of SARS-CoV-2 and SARS-CoV. Science.

[33] Winkler ES (2020). SARS-CoV-2 infection of human ACE2-transgenic mice causes severe lung inflammation and impaired function. Nat. Immunol..

[34] Oladunni FS (2020). Lethality of SARS-CoV-2 infection in K18 human angiotensin-converting enzyme 2 transgenic mice. Nat. Commun..

[35] Lo M (2017). Effector-attenuating substitutions that maintain antibody stability and reduce toxicity in mice. J. Biol. Chem..

[36] Weisblum Y (2020). Escape from neutralizing antibodies by SARS-CoV-2 spike protein variants. Elife.

[37] Baum A (2020). Antibody cocktail to SARS-CoV-2 spike protein prevents rapid mutational escape seen with individual antibodies. Science.

[38] Zhou H (2019). Structural definition of a neutralization epitope on the N-terminal domain of MERS-CoV spike glycoprotein. Nat. Commun..

[39] Dussupt V (2020). Potent Zika and dengue cross-neutralizing antibodies induced by Zika vaccination in a dengue-experienced donor. Nat. Med..

[40] Wang L (2021). Ultrapotent antibodies against diverse and highly transmissible SARS-CoV-2 variants. Science.

[41] Hansen J (2020). Studies in humanized mice and convalescent humans yield a SARS-CoV-2 antibody cocktail. Science.

[42] Andreano E (2021). Extremely potent human monoclonal antibodies from COVID-19 convalescent patients. Cell.

[43] Jones BE (2021). The neutralizing antibody, LY-CoV555, protects against SARS-CoV-2 infection in nonhuman primates. Sci. Transl. Med..

[44] Sun Y (2021). Structure-based development of three- and four-antibody cocktails against SARS-CoV-2 via multiple mechanisms. Cell Res..

[45] Yan R (2021). Structural basis for bivalent binding and inhibition of SARS-CoV-2 infection by human potent neutralizing antibodies. Cell Res..

[46] Rappazzo CG (2021). Broad and potent activity against SARS-like viruses by an engineered human monoclonal antibody. Science.

[47] Atyeo C (2020). Distinct early serological signatures track with SARS-CoV-2 survival. Immunity.

[48] Gorman MJ (2021). Fab and Fc contribute to maximal protection against SARS-CoV-2 following NVX-CoV2373 subunit vaccine with Matrix-M vaccination. Cell Rep. Med..

[49] Yuan M (2020). Structural basis of a shared antibody response to SARS-CoV-2. Science.

[50] Brown EP (2012). High-throughput, multiplexed IgG subclassing of antigen-specific antibodies from clinical samples. J. Immunol. Methods.

[51] Tomaras GD (2008). Initial B-cell responses to transmitted human immunodeficiency virus type 1: virion-binding immunoglobulin M (IgM) and IgG antibodies followed by plasma anti-gp41 antibodies with ineffective control of initial viremia. J. Virol..

[52] Moore MJ (2004). Retroviruses pseudotyped with the severe acute respiratory syndrome coronavirus spike protein efficiently infect cells expressing angiotensin-converting enzyme 2. J. Virol..

[53] Kirchdoerfer RN (2018). Stabilized coronavirus spikes are resistant to conformational changes induced by receptor recognition or proteolysis. Sci. Rep..

[54] Zhou T (2020). Structure-based design with tag-based purification and in-process biotinylation enable streamlined development of SARS-CoV-2 spike molecular probes. Cell Rep..

[55] Ackerman ME (2011). A robust, high-throughput assay to determine the phagocytic activity of clinical antibody samples. J. Immunol. Methods.

[56] Fischinger S (2019). A high-throughput, bead-based, antigen-specific assay to assess the ability of antibodies to induce complement activation. J. Immunol. Methods.

[57] ter Meulen J (2006). Human monoclonal antibody combination against SARS coronavirus: synergy and coverage of escape mutants. PLoS Med..

[58] Davidson E, Doranz BJ (2014). A high-throughput shotgun mutagenesis approach to mapping B cell antibody epitopes. Immunology.

[59] Strong M (2006). Toward the structural genomics of complexes: crystal structure of a PE/PPE protein complex from *Mycobacterium tuberculosis*. Proc. Natl Acad. Sci. USA.

[60] McCoy AJ (2007). Phaser crystallographic software. J. Appl. Crystallogr..

[61] Emsley P, Cowtan K (2004). Coot: model-building tools for molecular graphics. Acta Crystallogr. D Biol. Crystallogr..

[62] Adams PD, Mustyakimov M, Afonine PV, Langan P (2009). Generalized X-ray and neutron crystallographic analysis: more accurate and complete structures for biological macromolecules. Acta Crystallogr. D Biol. Crystallogr..

[63] Otwinowski Z, Minor W (1997). Processing of X-ray diffraction data collected in oscillation mode. Methods Enzymol..

[64] Scheres SH (2012). RELION: implementation of a Bayesian approach to cryo-EM structure determination. J. Struct. Biol..

[65] Punjani A, Rubinstein JL, Fleet DJ, Brubaker MA (2017). cryoSPARC: algorithms for rapid unsupervised cryo-EM structure determination. Nat. Methods.

[66] Pettersen EF (2004). UCSF Chimera—a visualization system for exploratory research and analysis. J. Comput. Chem..

[67] Schmidt F (2020). Measuring SARS-CoV-2 neutralizing antibody activity using pseudotyped and chimeric viruses. J. Exp. Med..

