# Supplementary material for: Low-dose in vivo protection and neutralization across SARS-CoV-2 variants by monoclonal antibody combinations
Source: Nat Immunol. 2021 Oct 29;22(12):1503–14. doi: 10.1038/s41590-021-01068-z (PMC8642242; doi:10.1038/s41590-021-01068-z)
Supplement: Supplementary file 1 — Supplementary Tables 1 and 2 [file 41590_2021_1068_MOESM1_ESM.pdf]

nature portfolio

https://doi.org/10.1038/s41590-021-01068-z

Supplementary information

Low-dose in vivo protection and neutralization across SARS-CoV-2 variants by monoclonal antibody combinations

In the format provided by the authors and unedited

Supplementary Table 1. WRAIR NTD and RBD mAbs comparisons with previously characterized SARS-CoV-2 mAbs.

	mAb	VH gene	% SHM	DH gene	JH gene	CDRH3 length	CDR1	CDR2	VDJ junction	VL gene	IC50 (µg/ml)	Reference
NTD neutralizing antibodies	WRAIR-2004	1-2	1.7	3-10	5	11	GYTFTGYY	INPSSGGT	CATDGDDGENWFDPW	VK1D-16	0.027	this manuscript
	WRAIR-2025	1-24	4.8	2-15	5	12	GYTLVELS	FDPEDGET	CVTSQPFVSPNWFDPW	VL2-14	0.009	
	WRAIR-2035	1-24	2.7	5-18	5	12	GYTLPELS	FDPEDGKI	CATSPAVVERGWFDPW	VL2-8	0.053	
	WRAIR-2039	1-24	2.4	4-17	4	11	GYMLIELS	FDPEDAET	CTTGPAVTNRPADYW	VL1-40	0.006	
	WRAIR-2196	1-24	2.0	6-25	4	13	GYTLIELS	FDPEDAKT	CATTPAFMAAAGDFDYW	VL3-27	0.109	
	**WRAIR-2008**	1-24	2.0	1-20	6	19	GYTLTELS	FDPEDGET	CATAGAITGTPRNFYYYYGMDVW	VK2-24	0.017	
	**WRAIR-2037**	1-24	1.0	1-20	6	19	GYTLTELS	FDPEDAET	CATAGAITGTPTNYSYYYGMDFW	VK2-24	0.072	
	**4A8**	1-24	1.4	6-19	6	19	GYTLTELS	FDPEDGET	CATSTAVAGTPDLFDYYYGMDVW	VK2-24	0.017	26
	1-87	1-24	1.0	6-19	6	19	GYTLIELS	FDPEDAET	CATGIAVIGPPPSTYYYYGMDVW	VL2-14	0.086	8
	CM25	1-24	n/a	3-10	5	12	GYTLTELS	FDPEDGET	CATGPAVRRGSWFDPW	VL1-51	0.010*	27
	FC05	1-24	1.0	6-13	5	12	GYTLPEVA	FDPEDGET	CATTTPFSSSYWFDPW	VK1-17	0.114*	49
RBD neutralizing antibodies	WRAIR-2123	3-30	1.7	3-10	4	18	GFTFSSYA	LSYDGSNK	CARDGVSVTMVRGVIGPLCDYW	VK1-33	0.004	this manuscript
	WRAIR-2125	3-30	1.0	3-22	1	18	GFTFSSYG	ISYDGSNK	CAKDSPYYYDSSGYYPGYFQDW	VK1-39	0.017	
	WRAIR-2165	3-48	1.0	6-25	4	18	GFTFSSYS	ISSSSSTI	CARVARRDVAPATIAAYFLDYW	VK3-11	0.010	
	WRAIR-2173	4-39	6.0	2-2	5	21	GDSISSSDYS	IYYIKNT	CARERPPFDVVVVPAARPYNWFDPW	VL1-40	0.004	
	P5A-1B6	3-30	0.0	-	-	18	GFTFSSYA	ISYDGSNK	CARDGQAITMVQGVIGPPFDYW	VK1-33	0.253	45
	P2B-2F6	4-38	-	-	-	18	GYSISSGYY	IYHSGST	CARAVVGIVVVPAAGRRAFDIW	VL2-8	0.050	12
	CB6	3-66	1.0	3-10	4	11	GFTVSSNY	IYSGGST	CARVLPMYGDYLDYW	VK1-39	0.023	50
	C002	3-30	0.3	2-15	4	15	GFTFSIYG	ISYDGSNK	CAKEGRPSDIVVVVAFDYW	VK1-39	0.008	29
	CC12.1	3-53	-	-	6	9	GLTVSSNY	IYSGSTF	CARDLDVYGLDVW	VK1-9	0.019	48
	S2E12	1-58	-	-	3	14	GFTFTSSA	IVVGSGNT	CASPYCSGGSCSDGFDIW	VK3-20	0.006*	16
	COV2-2196	1-58	-	2-2	3	14	GFTFMSSA	IVIGSGNT	CAAPYCSSISCNDGFDIW	VK3-20	0.001	4
	A23-58.4	1-58	0.0	2-8	6	14	GFTFTSSA	IVVGSGNT	CAAPNCSNVVCYDGFDIW	VK3-20	0.003	40
	LY-CoV555	1-69	0.3	-	-	16	GGTFSNYA	IIPILGIA	CARGYYEARHYYYYYAMDVW	VK1-39	0.012	43
	REGN10933	3-11	-	-	-	11	GFTFSNYA	ISYDGSNK	CASGSDYGDYLLVYW	VK1D-33	0.006	41

Germline gene assignments and characteristics of WRAIR and previously structurally characterized mAbs were obtained with IgBlast. HCDR3 lengths are based on Kabat numbering scheme. The half inhibitory concentration (IC50) is indicated for the pseudotyped assay, when available, or the authentic virus assay (marked by an asterisk). NTD-directed 4A8-like and RBD-directed S2E12-like antibodies are bolded and underlined, respectively.

Supplementary Table 2. Crystallographic Data Collection and Refinement Statistics

	RBD-A WRAIR-2125 + SARS-CoV-2 RBD (PDB: 7N4L)	RBD-A WRAIR-2173 + SARS-CoV-2 RBD (PDB: 7N4J)	RBD-B WRAIR-2057 + SARS-CoV-2 RBD (PDB: 7N4I)	RBD-C WRAIR-2151 + SARS-CoV-2 RBD (PDB: 7N4M)
**Data collection and processing**				
Beamline (Detector)	24-ID-E (Dectris Eiger 16M PIXEL)	19-ID (Q315r CCD)	24-ID-C (Dectris Eiger 16M PIXEL)	24-ID-E (Dectris Eiger 16M PIXEL)
Data processing	XDS	HKL2000 suite	HKL2000 suite	XDS
Search models	RBD[39], antibody 4-18 heavy chain (PDB code: 7L2E), human V-kappa domain light chain (PDB code: 2BX5)	RBD[39], Influenza mAb 6649 (PDB code: 5W6C)	RBD[39], transglutaminase 2-specific mAb (PDB code: 4XD3)	RBD[39], anti-EGFR mAb 059-152 (PDB code: 5XWD)
Space group	$P\ 6_1$	$P\ 2_1$	$P\ 4_3$	$P\ 4_3\ 2_1\ 2$
Cell dimensions				
a,b,c (Å)	188.9,188.9,65.08	46.26,70.46,107.25	73.93,73.93,149.18	112.91,112.91,239.50
α,β,γ (°)	90.0,90.0,120.0	90.0,98.2,90.0	90.0,90.0,90.0	90.0,90.0,90.0
Resolution (Å)	163.6-3.77 (4.27-4.0, 3.99-3.77)	50.0-2.2 (2.28-2.20)	50.0-2.28 (2.46-2.28)	119.75-3.85 (4.35-4.08,4.07-3.85)
R_{sym}	20.8 (57.4, 86.3)	14.9 (50.2)	10.6 (39.2)	17.6 (100.9, 213.6)
I / σI	4.05 (1.63, 0.95)	10.6 (3.6)	19.0 (3.1)	8.98 (2.46,1.07)
Reflections (unique/total)	24,288/43,744	34,103/219,115	36,098/183,662	27,912/199,543
Completeness (%)	93.0 (97.4, 78.6)	99.2 (98.8)	99.3 (93.8)	99.8 (100.0, 99.4)
Redundancy	1.8 (1.85, 1.63)	6.4 (5.5)	5.1 (4.1)	7.15 (7.34, 7.18)
$CC_{1/2}$	0.97 (0.67, 0.43)	0.95 (0.87)	0.99 (0.87)	0.99 (0.80, 0.42)
R_{pim}	19.7 (49.0, 84.2)	6.2 (23.3)	5.1 (20.2)	8.9 (32.5, 74.8)
**Refinement**				
Resolution (Å)	20.0-3.78	44.4-2.2	19.87-2.28	20.0-3.85
No. reflections	11,972	33,711	35,988	15,274
R_{work} / R_{free} *	0.26/0.30	0.17/0.20	0.20/0.23	0.25/0.29
Ramachandran Favoured/allowed/outliers	92.0/8.0/0.0	95.0/5.0/0.0	94.0/6.0/0.0	94.0/6.0/0.0
B-Factor				
Protein	66.5	43.7	53.7	207.3
R.m.s deviations				
Bond lengths (Å)	0.003	0.003	0.005	0.004
Bond angles (°)	0.678	0.703	0.755	0.651

Values in parentheses are for the highest-resolution shells. Values up to 2 high resolution shells are presented for WRAIR-2125 and WRAIR-2151 complex data sets. *R_{free} was calculated using ~5% randomly selected reflections.
