# Supplementary material for: Low-dose in vivo protection and neutralization across SARS-CoV-2 variants by monoclonal antibody combinations
Source: Nat Immunol. 2021 Oct 29;22(12):1503–14. doi: 10.1038/s41590-021-01068-z (PMC8642242; doi:10.1038/s41590-021-01068-z)
Supplement: Supplementary file 2 — Reporting Summary [file 41590_2021_1068_MOESM2_ESM.pdf]

nature research

Corresponding author(s): Shelly J. Krebs and M. Gordon Joyce

Last updated by author(s): Jun 4, 2021

Reporting Summary

Nature Research wishes to improve the reproducibility of the work that we publish. This form provides structure for consistency and transparency in reporting. For further information on Nature Research policies, see our Editorial Policies and the Editorial Policy Checklist.

Statistics

For all statistical analyses, confirm that the following items are present in the figure legend, table legend, main text, or Methods section.

n/a	Confirmed	
☐	☒	The exact sample size (*n*) for each experimental group/condition, given as a discrete number and unit of measurement
☐	☒	A statement on whether measurements were taken from distinct samples or whether the same sample was measured repeatedly
☐	☒	The statistical test(s) used AND whether they are one- or two-sided<br>*Only common tests should be described solely by name; describe more complex techniques in the Methods section.*
☒	☐	A description of all covariates tested
☒	☐	A description of any assumptions or corrections, such as tests of normality and adjustment for multiple comparisons
☐	☒	A full description of the statistical parameters including central tendency (e.g. means) or other basic estimates (e.g. regression coefficient) AND variation (e.g. standard deviation) or associated estimates of uncertainty (e.g. confidence intervals)
☐	☒	For null hypothesis testing, the test statistic (e.g. *F*, *t*, *r*) with confidence intervals, effect sizes, degrees of freedom and *P* value noted<br>*Give P values as exact values whenever suitable.*
☒	☐	For Bayesian analysis, information on the choice of priors and Markov chain Monte Carlo settings
☒	☐	For hierarchical and complex designs, identification of the appropriate level for tests and full reporting of outcomes
☒	☐	Estimates of effect sizes (e.g. Cohen's *d*, Pearson's *r*), indicating how they were calculated

Our web collection on statistics for biologists contains articles on many of the points above.

Software and code

Policy information about availability of computer code

Data collection	Diffraction data were collected at Advanced Photon Source (APS), Argonne National Laboratory beamlines. Diffraction data were collected at Advanced Photon Source (APS) beamlines. Diffraction data for WRAIR-2125-RBD and WRAIR-2151-RBD complexes were significantly anisotropic and were corrected using the UCLA Diffraction Anisotropy Server 73. All the crystal structures described in this study were solved by molecular replacement (MR) using PHASER, and iterative model building, and refinement were performed in COOT and Phenix74, 75, 76. Diffraction data quality was assessed using Phenix xtriage. Data collection, MR search models and refinement statistics are reported in Extended Data Table 2. All structures were refined using Phenix refine with positional, global isotropic B-factor refinement and defined TLS groups. Manual model building was performed in COOT. Overall, the Ramachandran plot as determined by MOLPROBITY showed 92-95% of all residues in favored regions and 4-6% of all residues in the allowed regions. Electron density for the structures was clearly interpretable except for the heavy chain Fc1 domain of WRAIR-2151. Interactive surfaces were analyzed using PISA (www.ebi.ac.uk/pdbe/pisa/) and are provided in Supplementary Table 1. Structure figures were prepared using PyMOL (DeLano Scientific).
Data analysis	Statistical analyses were performed in Prism (version 9, GraphPad Software), N-Parameter Logistic Regression (nplr) R package version 0.1-7, R (version 3.6.3) and R studio (1.2.1355). Data were graphed using Prism software (version 9, GraphPad Software). Flow plots were generated using FlowJo version 10 Real-time interactions between purified E proteins and antibodies were measured by Biolayer interferometry and analyzed by the ForteBio Data Analysis software 10.0. Structural diffraction data indexing, integration, and scaling were carried out using the HKL2000 suite37. Phenix xtriage (version 1.11.1-2575-0000) was used to analyze all the scaled diffraction data output from HKL2000 (suite 37) and XDS (version Jan 26, 2018 BUILT=20180126), and structure quality was assessed with MolProbity (Phenix suite version 1.11.1-2575-0000). All crystal structures described in this study were solved by molecular replacement using the program Phaser, version 2.139. All structure figures were generated using PyMOL (version 1.3).

For manuscripts utilizing custom algorithms or software that are central to the research but not yet described in published literature, software must be made available to editors and reviewers. We strongly encourage code deposition in a community repository (e.g. GitHub). See the Nature Research guidelines for submitting code & software for further information.

Data

Policy information about availability of data

All manuscripts must include a data availability statement. This statement should provide the following information, where applicable:

- Accession codes, unique identifiers, or web links for publicly available datasets
- A list of figures that have associated raw data
- A description of any restrictions on data availability

The associated data for the crystallographic complexes reported in this paper are available from the Protein Data Bank (PDB) with accession codes PDB: 7N4L, 7N4J, 7N4I and 7N4M. The antibody sequences are available at Genbank with accession numbers MZ825470-MZ825529. The source data that support the findings of this study are published alongside the paper, or available from the corresponding authors upon request.

Field-specific reporting

Please select the one below that is the best fit for your research. If you are not sure, read the appropriate sections before making your selection.

- [x] Life sciences
- [ ] Behavioural & social sciences
- [ ] Ecological, evolutionary & environmental sciences

For a reference copy of the document with all sections, see nature.com/documents/nr-reporting-summary-flat.pdf

Life sciences study design

All studies must disclose on these points even when the disclosure is negative.

Sample size	Monoclonal antibodies were isolated from 1 individual (Donor #3), who had the highest SARS-CoV-2 pseudotyped neutralization titer within the COVID-19 convalescent cohort RV229H. Passive transfer studies of monoclonal antibodies prophylactically or therapeutically were used to assess protection and survival from SARS-CoV-2 infection, including weight loss and viral replication in the lungs using the lethal transgenic K18 mouse model. Five independent experiments were conducted with using groups of 15 mice per group and compared to isotype controls within each experiment.
Data exclusions	No data were excluded from this analysis.
Replication	Binding and neutralization data were calculated from two independent experiments, performed in triplicate. Since there is inherent variability in different neutralization assays, 2 different types of neutralization assays (pseudtyped and live authentic virus PRNT) were performed to confirm the observations. Neutralization potencies obtained from each assay correlated for all mAbs tested, and all findings were replicated. The passive protection experiments were performed multiple in independent experiments with 15 mice per group using different doses ranging from 20 mg kg-1 down to 0.625 mg kg-1.
Randomization	A history of SARS-CoV-2 infection was a prerequisite for enrolling in RV229H, with samples collected between 6-8 weeks from symptom onset. Connvalescent plasma of 56 SARS-CoV-2- infected human donors, who had mild to moderate symptoms were tested for neutralization potency, and the donor (Donor #3) with potent plasma neutralizing antibodies against SARS-CoV-2 was selected for isolation of monoclonal antibodies. Randomization was not performed. For the in vivo mouse passive protection studies, mice were randomly assigned either treatment or sham groups for each study.
Blinding	The Principle Investigators were blinded to patient enrollment during the development of the convalescent cohorts. Samples from Donor #3 were chosen for monoclonal antibody isolation based upon high neutralization compared to other donors using the same criteria for enrollment. The Principle Investigators were also blinded to the treatment and sham groups for the in vivo mouse passive protection studies until the studies were complete and results were available.

Reporting for specific materials, systems and methods

We require information from authors about some types of materials, experimental systems and methods used in many studies. Here, indicate whether each material, system or method listed is relevant to your study. If you are not sure if a list item applies to your research, read the appropriate section before selecting a response.

Materials & experimental systems

n/a	Involved in the study	
[ ]	[x]	Antibodies
[ ]	[x]	Eukaryotic cell lines
[x]	[ ]	Palaeontology and archaeology
[ ]	[x]	Animals and other organisms
[ ]	[x]	Human research participants
[x]	[ ]	Clinical data
[x]	[ ]	Dual use research of concern

Methods

n/a	Involved in the study	
[x]	[ ]	ChIP-seq
[ ]	[x]	Flow cytometry
[x]	[ ]	MRI-based neuroimaging

Antibodies

Antibodies used

Antibodies not described in this study for the first time and their sources are as follows: mAbs CC12.1, CC12.16 (Rogers, et al, 2020) and CR30229 (Joyce, et al, 2020) were used to divide WRAIR RBD neutralizing mAbs into 3 distinct groups: RBD-A, B and C, respectively. The MM43 monoclonal antibody (SinoBiological, #40591-MM43) was used for primary staining of Spike ferritin nanoparticle (SpFN) antigen-specific B cells. For Flow cytometry: Mouse anti-human IgG, HRPconjugated (Southern Biotech, Catalog 9040-05, Lot J3314.T085 and L0717-ZC27B, Clone JDC-10, Dilution 1/2800), Goat antimouse IgG, HRP conjugated (Southern Biotech, Catalog 1030-05, Lot K3515-T566E, Dilution 1/6000), Mouse anti-human CD3, BV510 conjugated (BD Biosciences Cat# 563918, Lot 7037566, Clone SP34-2, Dilution 1/20), Mouse anti-human CD4, BV510 conjugated (BD Biosciences Cat# 562970, Lot 7094727, Clone SK3, Dilution 1/80), Mouse anti-human CD8, BV510 conjugated (BioLegend Cat# 301047, Lot B221676, Clone RPA-T8, Dilution 1/80), Mouse anti-human CD14, BV510 conjugated (BioLegend Cat# 301841, Lot B236875, Clone M5E2, Dilution 1/80), Mouse anti-human CD16, BV510 conjugated (BD Biosciences Cat#563830, Lot 7103547, Clone 3G8, Dilution 1/160), Mouse anti-human CD56, BV510 conjugated (BioLegend Cat# 318339, Lot B205718, Clone HCD56, Dilution 1/40), Mouse anti-human CD19, ECD conjugated (Beckman Coulter Cat# IM2708U, Lot 97, Clone J3-119, Dilution 1/40), Mouse anti-human IgG, BV785 conjugated (BD Biosciences Cat# 564230, Lot 7037913, Clone G18-145, Dilution 1/80), Mouse anti-human IgD, APC-Cy7 conjugated (BioLegend Cat# 348217, Lot B234185, Clone IA6-2, Dilution 1/160), Mouse anti-human IgM, PE-Cy5 conjugated (BD Biosciences Cat# 551079, Lot 7041529, Clone G20-127, Dilution 1/10).

Validation

All antibodies from commercial sources undergo validation using flow cytometry, western blot, chromatin immunoprecipitation, immunofluorescence, immunohistochemistry, and/or biofunctional assays to ensure specificity and to provide clarity for research uses. Specifically, mouse anti-human CD3, BV510 conjugated (Clone SP34-2), mouse anti-human CD16, BV510 conjugated (Clone 3G8), mouse anti-human IgG, BV785 conjugated (Clone G18-145), mouse anti-human IgM, PE-Cy5 conjugated (Clone G20-127) and mouse anti-human CD4, BV510 conjugated (Clone SK3) underwent stringent testing and validation by BD Biosciences to assure that it generates a high-quality conjugate with consistent performance and specific binding activity. The validation process included testing on a combination of primary cells, cell lines and/or transfectant cell models with relevant controls using multiple immunoassays to ensure biological accuracy. BD also performs multiplexing with additional antibodies to interrogate antibody staining in multiple cell populations. Mouse anti-human CD8, BV510 conjugated (Clone RPA-T8), mouse anti-human CD14, BV510 conjugated (Clone M5E2), mouse anti-human CD56, BV510 conjugated (Clone HCD56) and mouse antihuman IgD, APC-Cy7 conjugated (Clone IA6-2) were thoroughly validated by Biolegend. Each antibody was tested using the following criteria: 1.Staining of 1-3 target cell types with either single- or multi-color analysis detailed in the QC specification (including positive and negative controls). The tested cells can be primary cells and/or cell lines known to be positive or negative for the target antigen; 2.Each batch product is validated by QC testing with a series of dilutions to make sure the product is working within expected antibody titer range; 3.Each batch is compared to an internally established "gold standard" to maintain batch-to-batch consistency; 4. When applicable, our products are side-by-side tested with our competitors' products to make sure that BioLegend's products exceed or are at least the same quality; 5.For most tandem dye-conjugated products, color compensation is examined in order to verify tandem integrity.
General statements from Biolegend:
"The specificity and sensitivity of each antibody is thoroughly validated in the New Product Development stage. This is done by staining multiple target cells with either single- or multi-color analysis or by other testing approaches. The QC specifications and testing SOPs and gold standard for each product are then developed".
In general, each product is tested using the following criteria:
1. Staining of 1-3 target cell types with either single- or multi-color analysis detailed in the QC specification (including positive and negative controls). The tested cells can be primary cells and/or cell lines known to be positive or negative for the target antigen.
2. Each batch product is validated by QC testing with a series of dilutions to make sure the product is working within expected antibody titer range.
3. Each batch is compared to an internally established "gold standard" to maintain batch-to-batch consistency.

Eukaryotic cell lines

Policy information about cell lines

Cell line source(s)

HEK293T/17 (ATCC #CRL-11268), HEK293-ACE2 (Integral Molecular), Vero E6 (ATCC #CRL-1586), Expi293F (ThermoFisher Scientific #A14527), Freestyle 293F (ThermoFisher Scientific #R79007), THP-1 (Millipore #88081201) cell lines were utilized in this study.

Authentication

All cell lines were authenticated using short-tandem repeat analysis.

Mycoplasma contamination

Cell lines were not recently tested for mycoplasma contamination.

Commonly misidentified lines
(See ICLAC register)

No commonly misidentified cell lines were used in this study.

Animals and other organisms

Policy information about studies involving animals; ARRIVE guidelines recommended for reporting animal research

Laboratory animals

Eight- to ten-week old K18-hACE2 transgenic mice were obtained from Jackson Laboratories (Bar Harbor, ME) were used in this study. Mice were randomized evenly per group between male and female mice, with a total of 15 mice used per group, and were housed within normal parameters of dark/light cycle, ambient temperature and humidity.

Wild animals

This study did not involve wild animals.

Field-collected samples | This study did not involve samples collected from the field.

Ethics oversight | This study was approved by the Institutional Animal Care and Use Committees (IACUCs) at both The Trudeau Institute and U.S. Army Medical Research. Research was conducted in compliance with the Animal Welfare Act, and other federal statutes and regulations relating to animals and experiments involving animals and adhered to the ethical principles stated in the Guide for the Care and Use of Laboratory Animals, NRC Publication, 1996 edition.

Note that full information on the approval of the study protocol must also be provided in the manuscript.

Human research participants

Policy information about studies involving human research participants

Population characteristics | RV229H enrolled men and women, ages 30 to 65 years, with previous documented SARS-CoV-2 infection. Participants had a range of symptoms from mild to severe, but fully recovered by enrollment and sample collection. Donor #3, aged 60 years had a range of symptoms from mild to moderate. PBMCs were collected 6 weeks following symptom onset and were used to isolate monoclonal antibodies.

Recruitment | Men and women, ages 30 to 65 years, with documented SARS-CoV-2 infection following 4-10 weeks of were recruited to provide blood samples (plasma and PBMCs). Participants had a range of symptoms from mild to severe, but fully recovered by enrollment and sample collection.

Ethics oversight | These studies were approved by the Walter Reed Army Institute of Research (WRAIR) Institutional Review Board and written informed consent was obtained from all participants.

Note that full information on the approval of the study protocol must also be provided in the manuscript.

Flow Cytometry

Plots

Confirm that:

☒ The axis labels state the marker and fluorochrome used (e.g. CD4-FITC).

☒ The axis scales are clearly visible. Include numbers along axes only for bottom left plot of group (a 'group' is an analysis of identical markers).

☒ All plots are contour plots with outliers or pseudocolor plots.

☒ A numerical value for number of cells or percentage (with statistics) is provided.

Methodology

Sample preparation | Cryopreserved PBMCs were thawed in warm media containing benzonase, then washed with PBS and stained for viability using the Aqua Live/Dead stain (ThermoFisher). Cells were incubated at 21°C for 30min with a cocktail of antibodies including CD3 BV510 (BioLegend), CD4 BV510 (BD Biosciences), CD8 BV510 (BioLegend), CD14 BV510 (BioLegend), CD16 BV510 (BD Biosciences) and CD56 BV510 (BioLegend) as dump channel markers, and CD19 PE Dazzle 594 (BioLegend), CD38 BUV496 (BD Biosciences), CD27 BV605 (BioLegend), CD20 AF700 (BD Biosciences), IgD APC/Cyanine7 (BioLegend), integrin [illegible]7 PE/Cyanine7 (BD Biosciences), IgG (BioLegend), CD10 BUV395 (BD Biosciences), CD21 FITC (BioLegend), and IgM BV650 (BioLegend). Two sorting strategies were used to maximize the number of probes used to isolate antigen-specific B cells: The first strategy utilized a stabilized SARS-CoV-2 S trimer (HexaPro21) conjugated to streptavidin-APC, and the second strategy utilized a multivalent spike ferritin nanoparticle (SpFN28) displaying eight S trimers to potentially capture conformation-specific B cell receptors. SpFN was incubated with cells during primary staining, and SpFN+ B cell were identified by secondary staining using the MM43 monoclonal antibody (SinoBiological, #40591-MM43) conjugated to AF647 (ThermoFisher). Both strategies included SARS-CoV-2 RBD, S1, and S2 (ThermoFisher) which were biotinylated, tetramerized, and conjugated to streptavidin-PE. Since these antigens used the same conjugated streptavidin-PE, B cell binding could not be distinguished between SARS-CoV-2 RBD, S1, and S2 using flow cytometry. Specific B cell binding by flow cytometry was determined to the stabilized trimer using conjugated APC, and SpFN using AF647 conjugated to MM43. CD19+ B Cells that were antigen-specific were single-cell sorted into PCR plates containing lysis buffer composed of murine RNAse inhibitor (New England Biolabs), dithiothreitol (DTT), SuperScript III First Strand Buffer (ThermoFisher), Igepal (Sigma), and carrier RNA (Qiagen) at one cell per well using a FACS ARIA (Becton Dickinson) and stored at -80°C until subsequent reverse transcription.

Instrument | FACSAria (Becton Dickinson)

Software | FlowJo, version 10

Cell population abundance | Less than 5% of the total B cell population was specific for SARS-CoV-2 antigens or spike ferritin nanoparticles (SpFN). All antigen- positive B cells were sorted into lysis buffer, sequenced, cloned and characterized.

Gating strategy

CD19+/IgG+/IgD-/IgM- B cells reactive to SARS-CoV-2 antigens, or SpFN particles were sorted directly into lysis buffer at one cell per well into PCR plates using a FACSAria (Becton Dickinson) and stored at −80°C until subsequent reverse transcription. Increased frequencies of antigen-specific and cross-reactive B cells were detected in Donor #3, following SARS-CoV-2 infection compared to pre-pandemic PBMC samples. Gating strategy was supplied in Extended Data Fig. 1.

☒ Tick this box to confirm that a figure exemplifying the gating strategy is provided in the Supplementary Information.
