# Supplementary material for: Low-dose in vivo protection and neutralization across SARS-CoV-2 variants by monoclonal antibody combinations
Source: Nat Immunol. 2021 Oct 29;22(12):1503–14. doi: 10.1038/s41590-021-01068-z (PMC8642242; doi:10.1038/s41590-021-01068-z)
Supplement: Supplementary file 3 — Peer Review Information [file 41590_2021_1068_MOESM3_ESM.pdf]

natureresearch

# Peer Review Information

**Journal:** Nature Immunology
**Manuscript Title:** Low-dose *in vivo* protection and neutralization across SARS-CoV-2 variants by monoclonal antibody combinations
**Corresponding author name(s):** M. Gordon Joyce and Shelly J. Krebs

## Editorial Notes:

| | |
|---|---|
| **Transferred manuscripts** | This manuscript has been previously reviewed at another journal that is not operating a transparent peer review scheme. This document only contains reviewer comments, rebuttal and decision letters for versions considered at Nature Immunology. |
| **Redactions – unpublished data** | Parts of this Peer Review File have been redacted as indicated to maintain the confidentiality of unpublished data. |

## Reviewer Comments & Decisions:

**Decision Letter, initial version:**

**Subject:** Decision on Nature Immunology submission NI-LE32345-T
**Message:** 20th Jul 2021

Dear Dr Krebs,

Thank you for providing a point-by-point response to the refereess' comments on your manuscript entitled, "Low-dose in vivo protection and coverage across SARS-CoV-2 variants by monoclonal antibody combinations". As noted previously, they find your work of interest, some important points are raised, which appears that you and your colleagues are prepared to quickly respond and/or already have data in hand to address these concerns. We are very interested in the possibility of publishing your study in Nature Immunology, but would like to you to submit a revised manuscript before we make a final decision on publication.

We therefore invite you to revise your manuscript taking into account all reviewer and editor comments. Please highlight all changes in the manuscript text file in Microsoft Word format.

We are committed to providing a fair and constructive peer-review process. Do not hesitate to contact us if there are specific requests from the reviewers that you believe are

technically impossible or unlikely to yield a meaningful outcome.

When revising your manuscript:

* Include a “Response to referees” document detailing, point-by-point, how you addressed each referee comment. If no action was taken to address a point, you must provide a compelling argument. This response will be sent back to the referees along with the revised manuscript.

* If you have not done so already please begin to revise your manuscript so that it conforms to our Letter format instructions at http://www.nature.com/ni/authors/index.html. Refer also to any guidelines provided in this letter.

* Please include a revised version of any required reporting checklist. It will be available to referees to aid in their evaluation of the manuscript goes back for peer review. They are available here:

Reporting summary:
https://www.nature.com/documents/nr-reporting-summary.pdf

When submitting the revised version of your manuscript, please pay close attention to our href="https://www.nature.com/nature-research/editorial-policies/image-integrity">Digital Image Integrity Guidelines.</a> and to the following points below:

-- that unprocessed scans are clearly labelled and match the gels and western blots presented in figures.
-- that control panels for gels and western blots are appropriately described as loading on sample processing controls
-- all images in the paper are checked for duplication of panels and for splicing of gel lanes.

Finally, please ensure that you retain unprocessed data and metadata files after publication, ideally archiving data in perpetuity, as these may be requested during the peer review and production process or after publication if any issues arise.

Please use the link below to submit your revised manuscript and related files:
[REDACTED]

<strong>Note:</strong> This URL links to your confidential home page and associated information about manuscripts you may have submitted, or that you are reviewing for us. If you wish to forward this email to co-authors, please delete the link to your homepage.

We hope to receive your revised manuscript within one month. If you cannot send it within this time, please let us know. We will be happy to consider your revision so long as nothing similar has been accepted for publication at Nature Immunology or published elsewhere.

Please do not hesitate to contact me if you have any questions or would like to discuss these revisions further.

Nature Immunology is committed to improving transparency in authorship. As part of our efforts in this direction, we are now requesting that all authors identified as ‘corresponding author’ on published papers create and link their Open Researcher and Contributor Identifier (ORCID) with their account on the Manuscript Tracking System (MTS), prior to acceptance. ORCID helps the scientific community achieve unambiguous attribution of all scholarly contributions. You can create and link your ORCID from the home page of the MTS by clicking on ‘Modify my Springer Nature account’. For more information please visit please visit <a href="http://www.springernature.com/orcid">www.springernature.com/orcid</a>.

We look forward to seeing the revised manuscript and thank you for the opportunity to review your work.

Kind regards,

Laurie

Laurie A. Dempsey, Ph.D.
Senior Editor
Nature Immunology
l.dempsey@us.nature.com
ORCID: 0000-0002-3304-796X

Referee expertise:

Referee #1: Anti-viral B cell responses

Referee #2: Viral infectious disease

Referee #3: Structural biology

Referee #4: Antibody interactions

Reviewers' Comments:

Reviewer #1:
Remarks to the Author:
Neutralizing antibodies were isolated from a SARS-CoV-2 infected individual using a WT Spike protein or Spike displayed on ferritin particle. Abs binding either NTD, RBD and S2 were isolated and 117 were expressed for characterisation. Neutralizing Abs were mostly found against RBD and NTD. NTD nAbs were SARS-CoV-2 specific whereas some RBD nAbs also neutralized SARS-CoV-1. The authors examined the neutralizing properties, effector function activity, structure, and escape mechanisms. The authors use the K18-hACE2 transgenic SARS-CoV-2 mouse model to assess the ability of SARS-CoV-2 monoclonal antibodies to prevent infection and as a therapy, and examine the role of Fc in these models. They also examine how mAbs against different epitopes can improve

activity in the challenge models and minimize viral escape. Finally, they measure activity against SARS-CoV-2 viral variants.

This is an extremely comprehensive study which adds important and interesting insights to what has previously been reported by other groups in this area.

The paper is very clearly written, the methods described in appropriate detail and the experiments carried out to a high standard.

Minor points:

Reference to other papers showing low neutralization plateau for NTD nAbs should be included.

Why do the authors think NTD mAbs are better at facilitating complement recruitment?

How do the epitopes on the NTD nAbs relate to those already reported?

Data points should be included on figure 3b.

Extended figure 3c – it would be helpful to have the IC50 values as well as the fold change so that can know if the Fab's retain any neutralizing activity.

Extended figure 4b and 5e might be easier to see in a table format.

The title is a bit misleading as it could be read that the protection studies relate to the SARS-CoV-2 variants but the variants were only used in neutralization assays.

Reviewer #2:
Remarks to the Author:
In their manuscript Dussupt and colleagues describe novel mAbs against SARS-CoV-2 that target RBD and NTD. While many such mAbs have been reported, the manuscript describes many novelties including a focus on the NTD and the role of Fc-effector functions. However, there are several points that need the authors attention.

Major points

1) The authors need to distinguish between entry inhibition (pseudotyped viruses) and neutralization (authentic virus). Only one assay was done with authentic virus, everything else is based on entry inhibition.

2) It would be good to include activity against B.1.617.2 of the mAbs.

3) Almost all effector function measurements are non-quantitative. Actual dilution curves should be performed and the curves should be shown. One-point measurements for these assays are very much misleading.

Minor points

1) Many abbreviations including in the abstract and the methods section are not defined.

2) Throughout the manuscript (especially in some parts of the methods section (e.g. lines 1114-1120) and the abstract) words start with capital letters mid-sentence without any reason.

3) Line 163: 'SARS-CoV-2', not 'SARS-COV-2'

4) Line 334: 'the WRAIR-2125'

5) Line 377: remove 'disease'. That's what the D in COVID-19 stands for.

6) Line 840: 'BEI Resources'

7) Line 991: Isn't streptomycin usually specified in ug, not in units?

8) Line 1027: The reference is weirdly formatted.

Reviewer #3:
Remarks to the Author:
Since the initial outbreak of SARS-CoV-2, there have been a number of published studies on isolating monoclonal antibodies from convalescent donors. Although the authors report here a set of antibodies, very similar to what have been described in literature already, this study is probably one of the most comprehensive studies with some interesting details, such as properties of combined NTD and RBD antibodies, as well as how they may react with circulating variants of concerns. The paper is unusually long, nonetheless well-written, and packed with an enormous amount of data from biochemistry, structural biology to immunology and animal challenge studies. In general, the data are technically sound and convincing; the findings remain significant to the fight against the COVID-19 pandemic. There are only a few minor comments.

Line 55, "While initial isolation efforts primarily focused on RBD-directed antibodies in previous studies8-13". Not sure this is true since several published studies have isolated antibodies against the NTD, probably very similar to what are presented here. A supplemental table to summarize the similarities and differences between the antibodies described here and those in literature may help readers appreciate the novelty of the current study.

Line 74, "The majority of the mAbs bound to S2, followed by RBD and NTD, based on binding antibody assays (Fig. 1b)". It is unclear why most isolated antibodies are S2-directed, nonneutralizing ones. Did the donor mainly produce this type of antibodies or did it have to do with the sorting strategies? Indeed, a little more clarification on how exactly the cell sorting was performed may be helpful. As described in the main text and Methods, two different S trimer antigens were used, but how and why RBD, S1 and S2 antigens (mixed together?) were also used? S2 alone will probably fold into the postfusion conformation. Would using a postfusion reagent contribute to isolation of nonneutralizing antibodies?

The abstract may need to be further improved since it does not seem to capture all

important findings in the manuscript.

Reviewer #4:
Remarks to the Author:
Dussupt et al. generated several anti-NTD and anti-RBD neutralizing monoclonal antibodies from SARS-CoV2 infected individuals. They extensively analyzed the properties and functions of these neutralizing antibodies that recognize different epitopes. In particular, they used X-ray crystallography to determine the fine epitopes of these antibodies. They also showed that the use of both anti-NTD and anti-RBD-neutralizing antibodies enabled to neutralize SARS-CoV-2 infection with a low dose of antibodies using mouse model. Furthermore, they suggested that the combination of the two types of antibodies is effective against VOCs, which have recently become prevalent.

The experiments are well organized, and the results are clear and reasonable. The use of anti-RBD and anti-NTD neutralizing antibodies against SARS-CoV-2 infection is a new strategy and seems to be very effective in treating COVID-19. In addition, the authors suggested that anti-NTD antibodies they established were effective to VOCs by demonstrating that their anti-NTD antibodies were effective against the B.1.351 L242H variant. However, more than 99% of the current B.1.351 is the 241-243del type, and their anti-NTD antibody was not effective at all against B.1.351 241-243del type. This means that the combination of anti-NTD antibodies and anti-RBD antibodies cannot be used practically against the all current VOCs. The only effective combination for VOCs was the one of 2125 and 2151 antibodies, both of which recognize RBD. However, similar anti-RBD antibody combinations are already known. I understand that NTD/RBD mAb combinations are effective to the wild-type SARS-CoV-2. However, unfortunately, wild-type SARS-CoV-2 is no longer detected. Therefore, I do not agree with their main conclusion that "NTD/RBD mAb combinations confer potent protection".

Considering that all VOCs escape from most of anti-NTD neutralizing antibodies, anti-NTD neutralizing antibodies seem to be quite important to control the SARS-CoV-2 infection. Therefore, the authors' concept to use both anti-NTD and anti-RBD is very important. Similar anti-NTD neutralizing antibodies have already been reported in several papers, but as far as I know, none of them work on VOCs. I hope the authors could find anti-NTD neutralizing antibodies that work for VOCs. If the authors could identify anti-NTD antibodies that recognize the current VOCs, the antibodies will be very powerful to protect SARS-CoV-2 by combining with anti-RBD antibodies that can recognize VOCs such as 2125 antibody.

1. It would be better to show the effect on the Delta variant (B.1.617.2). If their anti-NTD neutralizing antibodies are effective to the Delta variant, their findings would be very much strengthened even if their antibodies do not work for B.1.1.7 or B.1.351 VOCs.

2. What is the molecular mechanism by which anti-NTD antibodies neutralize? Elucidating the molecular mechanism underlying the neutralization of SARS-CoV-2 by anti-NTD antibodies would provide much insight into this paper.

3. Many references lack page numbers.

**Author Rebuttal to Initial comments**

**Point by Point responses**

*We would like to thank all the reviewers for careful examination of the manuscript and pertinent and constructive comments that have helped improve the overall quality and clarity of the manuscript. Below, the reviewers' comments are bolded and our point-by-point responses are not bolded.*

**Reviewer #1**

**Neutralizing antibodies were isolated from a SARS-CoV-2 infected individual using a WT Spike protein or Spike displayed on ferritin particle. Abs binding either NTD, RBD and S2 were isolated and 117 were expressed for characterization. Neutralizing Abs were mostly found against RBD and NTD. NTD nAbs were SARS-CoV-2 specific whereas some RBD nAbs also neutralized SARS-CoV-1. The authors examined the neutralizing properties, effector function activity, structure, and escape mechanisms. The authors use the K18-hACE2 transgenic SARS-CoV-2 mouse model to assess the ability of SARS-CoV-2 monoclonal antibodies to prevent infection and as a therapy, and examine the role of Fc in these models. They also examine how mAbs against different epitopes can improve activity in the challenge models and minimize viral escape. Finally, they measure activity against SARS-CoV-2 viral variants.**

**This is an extremely comprehensive study which adds important and interesting insights to what has previously been reported by other groups in this area.**

**The paper is very clearly written, the methods described in appropriate detail and the experiments carried out to a high standard.**

We sincerely appreciate these positive comments.

**Minor points:**

**Reference to other papers showing low neutralization plateau for NTD nAbs should be included.**

We thank this reviewer for raising this point. We added references accordingly on page 5 to the following statement: "NTD mAbs displayed potent neutralization in both assays, with a notable difference: neutralization curves plateaued at around 75% neutralization in the pSV assay, as previously observed, while the same NTD mAbs achieved 100% neutralization of authentic SARS-CoV-2 (Fig. 1e,f).[1,2]"

**Why do the authors think NTD mAbs are better at facilitating complement recruitment?**

This is an important question. We hypothesize that the angle of mAb binding to the NTD plays an important role in complement recruitment to the antibody Fc domain. The RBD epitope on the Spike is not always accessible for binding, as it typically switches between a fully closed trimer or a form with RBDs in the ‘up’ conformation. Conversely, the NTD epitope is consistently available for mAb binding. Our data also suggests that the NTD mAbs bind with higher affinity to the spike glycoprotein compared to RBD-targeting mAbs. In addition, the distance between the NTD domains on a single Spike molecule allows multiple antibodies to readily bind, while the proximity of the three RBD domains to each other on a single Spike trimer may reduce the accessibility of multiple molecules to bind the Spike, thus limiting the number of Fc domains per Spike. Therefore, we hypothesize that the strength of binding, as well as the angle of antibody binding play a role in facilitating complement recruitment by NTD neutralizing mAbs. We added statements to page 16-17, of the revised manuscript that incorporate these hypotheses.

**How do the epitopes on the NTD nAbs relate to those already reported?**

This is a great idea. Per this comment and the comment by Reviewer #3, we added Supplemental Table 1 summarizing the differences and similarities (e.g. Germline origin, %SHM, CDR H3 length, IC50 values) between antibodies reported here and previously published antibodies.

**Data points should be included on figure 3b**.

Thank you, we agree with this reviewer and added the data points to Figure 3b as well as Supplemental Figure 8.

**Extended figure 3c – it would be helpful to have the IC50 values as well as the fold change so that can know if the Fab’s retain any neutralizing activity.**

Thank you for this comment. We added the IC50 values to Supplemental Figure 3c to add clarity the fold change in neutralization.

**Extended figure 4b and 5e might be easier to see in a table format.**

As requested, we modified these graphs into a Table format for ease of understanding for the reader for both Supplemental Figures 4b and 5e. A heat map was used in each table to highlight the residues important for binding, as determined by loss of binding following alanine mutations at those particular sites.

**The title is a bit misleading as it could be read that the protection studies relate to the SARS-CoV-2 variants but the variants were only used in neutralization assays.**

We agree with this comment and changed the title to: "*Low-dose in vivo protection and neutralization across SARS-CoV-2 variants by monoclonal antibody combinations*" to specify neutralization of the variants.

**Reviewer #2**
**In their manuscript Dussupt and colleagues describe novel mAbs against SARS-CoV-2 that target RBD and NTD. While many such mAbs have been reported, the manuscript describes many novelties including a focus on the NTD and the role of Fc-effector functions. However, there are several points that need the authors attention.**

**Major points:**

**1) The authors need to distinguish between entry inhibition (pseudotyped viruses) and neutralization (authentic virus). Only one assay was done with authentic virus, everything else is based on entry inhibition.**

We thank this reviewer for this important comment, as even though these assays (pseudotyped neutralization and neutralization of authentic viruses) strongly correlate as shown in Figure 1g (rho=0.8155, p<0.0001), there are differences between these assays. As another reviewer pointed out, neutralization plateaus in the pseudotyped assay by mAbs targeting the NTD, while the same NTD-mAbs show complete neutralization in the authentic virus neutralization assay, similar to previously published studies[1, 2] . As noted by this reviewer, the pseudotyped assay largely demonstrates entry inhibition, especially from RBD-directed antibodies that block ACE-2 binding, as shown in Supplemental Figure 5a. As such, correlations between ACE-2 inhibition and pSV neutralization assays also strongly correlate. Nonetheless, pSV neutralization strongly correlates with authentic virus neutralization, is used widely as a surrogate for authentic virus neutralization (especially the absence of authentic virus assays for the variants of concern), and associates with *in vivo* protection in mRNA-1273 vaccinated animals[3]. The authentic neutralization experiments in this manuscript were performed in 2 independent assays, and pSV neutralization assays were performed in 2-3 independent assays. To sastify this comment, we have revised the manuscript to specify pSV neutralization versus authentic neutralization throughout the manuscript to make this important distinction.

**2) It would be good to include activity against B.1.617.2 of the mAbs.**

We agree! We anticipated that we would need to test these mAbs and mAb combinations against the Delta (B.1.617.2) variant, and have included binding and neutralization activity within the revised manuscript in Figure 4a. With the exception of WRAIR-2008, NTD-targeting mAbs bound to the B.1.617.2 Delta VOC similar to the original strain (WA1/2020), and 2 NTD mAbs modestly neutralized the Delta VOC at 1ug $mL^{-1.}$. All WRAIR RBD-targeting mAbs were able to bind and neutralize B.1.617.2, with WRAIR-2125 being the most potent with an IC50 of 3 ng $mL^{-1}$. This data has been added to the revised version of the manuscript in Figure 4 a-c.

**3) Almost all effector function measurements are non-quantitative. Actual dilution curves should be performed and the curves should be shown. One-point measurements for these assays are very much misleading.**

Thank you for this comment. We have added the dilution curves of these assays to Figure 3b, Supplemental Figure 3f, g and Supplemental Figure 8a to support our findings in the revised manuscript.

**Minor points**
**1) Many abbreviations including in the abstract and the methods section are not defined.**

We have defined these abbreviations in the abstract and **methods** in the revised manuscript.

**2) Throughout the manuscript (especially in some parts of the methods section (e.g. lines 1114-1120) and the abstract) words start with capital letters mid-sentence without any reason.**
**3) Line 163: 'SARS-CoV-2', not 'SARS-COV-2'**
**4) Line 334: 'the WRAIR-2125'**
**5) Line 377: remove 'disease'. That's what the D in COVID-19 stands for.**
**6) Line 840: 'BEI Resources'**
**7) Line 991: Isn't streptomycin usually specified in ug, not in units?**
**8) Line 1027: The reference is weirdly formatted.**

Minor comments #2-#7 have been corrected the in the revised manuscript where indicated. Of note, in some cases, software program names are capitalized. In addition, per this reviewer's comment and other reviewer comments, we have included additional references and reformatting corrections.

**Reviewer #3**
**Since the initial outbreak of SARS-CoV-2, there have been a number of published studies on isolating monoclonal antibodies from convalescent donors. Although the authors report here a set of antibodies, very similar to what have been described in literature already, this study is probably one of the most comprehensive studies with some interesting details, such as properties of combined NTD and RBD antibodies, as well as how they may react with circulating variants of concerns. The paper is unusually long, nonetheless well-written, and packed with an enormous amount of data from biochemistry, structural biology to immunology and animal challenge studies. In general, the data are technically sound and convincing; the findings remain significant to the fight against the COVID-19 pandemic. There are only a few minor comments.**

We sincerely thank this reviewer for their positive and encouraging comments.

**Line 55, "While initial isolation efforts primarily focused on RBD-directed antibodies in previous studies8-13". Not sure this is true since several published studies have isolated antibodies against the NTD, probably very similar to what are presented here. A supplemental table to summarize the similarities and differences between the antibodies described here and those in literature may help readers appreciate the novelty of the current study.**

This is a great idea. Per this comment and the comment by Reviewer #1, we added Supplemental Table 1 summarizing the differences and similarities (e.g. Germline origin, %SHM, CDR H3 length, IC50 values) between antibodies reported here and previously published antibodies.

**Line 74, "The majority of the mAbs bound to S2, followed by RBD and NTD, based on binding antibody assays (Fig. 1b)". It is unclear why most isolated antibodies are S2-directed, nonneutralizing ones. Did the donor mainly produce this type of antibodies or did it have to do with the sorting strategies? Indeed, a little more clarification on how exactly the cell sorting was performed may be helpful. As described in the main text and Methods, two different S trimer antigens were used, but how and why RBD, S1 and S2 antigens (mixed together?) were also used? S2 alone will probably fold into the postfusion conformation. Would using a postfusion reagent contribute to isolation of nonneutralizing antibodies?**

These are many excellent questions. In a separate [REDACTED]. Post-infection, the prevalence of convalescent donors targeting NTD, RBD, and S2 are similar when assessing binding antibodies to these domains (see graph below). However, the magnitude of binding to S2 was

significantly higher than binding to NTD or RBD (see below), suggesting that S2 targeting in SARS-CoV-2 infection may be a dominant target compared to NTD or RBD. The S2 antigen used in this study is not conformationally-constrained, and likely displays linear epitopes that are not in states of pre- or post-fusion conformations when used in sorting. To address this comment, we clarified the sorting strategy by providing greater detail in the methods, and added language regarding the potential influence of the sorting strategy on the isolation of S2 mAbs on page 4, line 136.

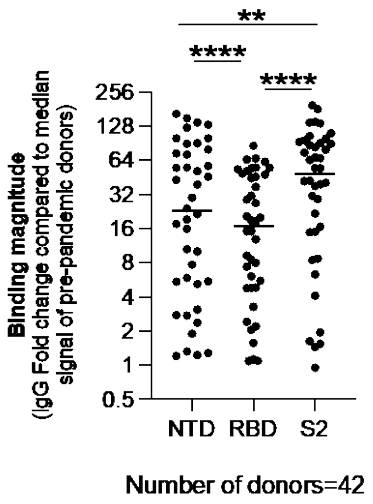


**The abstract may need to be further improved since it does not seem to capture all important findings in the manuscript.**

We agree that there are many important findings in this manuscript and have revised the abstract to reemphasize some of these salient points, such as VOC neutralization and Fc effector function. Given the word limit, we hope these revisions help capture more of the major highlights of the manuscript.

**Reviewer #4**

**Dussupt et al. generated several anti-NTD and anti-RBD neutralizing monoclonal antibodies from SARS-CoV2 infected individuals. They extensively analyzed the properties and functions of these neutralizing antibodies that recognize different epitopes. In particular, they used X-ray crystallography to determine the fine epitopes of these antibodies. They also showed that**

**the use of both anti-NTD and anti-RBD-neutralizing antibodies enabled to neutralize SARS-CoV-2 infection with a low dose of antibodies using mouse model. Furthermore, they suggested that the combination of the two types of antibodies is effective against VOCs, which have recently become prevalent.**

**The experiments are well organized, and the results are clear and reasonable. The use of anti-RBD and anti-NTD neutralizing antibodies against SARS-CoV-2 infection is a new strategy and seems to be very effective in treating COVID-19. In addition, the authors suggested that anti-NTD antibodies they established were effective to VOCs by demonstrating that their anti-NTD antibodies were effective against the B.1.351 L242H variant. However, more than 99% of the current B.1.351 is the 241-243del type, and their anti-NTD antibody was not effective at all against B.1.351 241-243del type. This means that the combination of anti-NTD antibodies and anti-RBD antibodies cannot be used practically against the all current VOCs. The only effective combination for VOCs was the one of 2125 and 2151 antibodies, both of which recognize RBD. However, similar anti-RBD antibody combinations are already known. I understand that NTD/RBD mAb combinations are effective to the wild-type SARS-CoV-2.**
**However, unfortunately, wild-type SARS-CoV-2 is no longer detected. Therefore, I do not agree with their main conclusion that "NTD/RBD mAb combinations confer potent protection".**

**Considering that all VOCs escape from most of anti-NTD neutralizing antibodies, anti-NTD neutralizing antibodies seem to be quite important to control the SARS-CoV-2 infection. Therefore, the authors' concept to use both anti-NTD and anti-RBD is very important. Similar anti-NTD neutralizing antibodies have already been reported in several papers, but as far as I know, none of them work on VOCs. I hope the authors could find anti-NTD neutralizing antibodies that work for VOCs. If the authors could identify anti-NTD antibodies that recognize the current VOCs, the antibodies will be very powerful to protect SARS-CoV-2 by combining with anti-RBD antibodies that can recognize VOCs such as 2125 antibody.**

**1. It would be better to show the effect on the Delta variant (B.1.617.2). If their anti-NTD neutralizing antibodies are effective to the Delta variant, their findings would be very much strengthened even if their antibodies do not work for B.1.1.7 or B.1.351 VOCs.**

We thank this reviewer for these important comments. We agree that the dominant circulating strains in many parts of the world are now variants of concern that have become more prevalent overtime. Since the time of this manuscript submission, the dominant strain in the US has become the Delta variant of concern (B.1.671.2), accounting for over 90% of new infections,

while the previous strain has decreased in prevalence. We anticipated that we would need to test these mAbs and mAb combinations against the Delta (B.1.617.2) variant, and have included binding and neutralization activity within the revised manuscript in Figure 4a. NTD-targeting mAbs bound to the B.1.617.2 VOC similar to the original strain, and 2 NTD mAbs modestly neutralized the Delta VOC at 1ug $mL^{-1.}$. All WRAIR RBD-targeting mAbs were able to potently neutralize B.1.617.2, with WRAIR-2125 being the most potent with an IC50 of 3 ng $mL^{-1}$. This data has been added to the revised version of the manuscript in Figure 4 a-c and discussed in the text starting on page 13.

**2. What is the molecular mechanism by which anti-NTD antibodies neutralize? Elucidating the molecular mechanism underlying the neutralization of SARS-CoV-2 by anti-NTD antibodies would provide much insight into this paper.**

Three studies on NTD-targeting antibodies have extensively examined the possible mechanisms of neutralization of this class of antibodies[2, 4, 5]. Our data suggest that our NTD-targeting antibodies are similar to the these previously described. Our data agrees with their conclusions that NTD neutralizing antibodies target the "NTD antigenic supersite" epitope on spike with similar angles of approach. NTD-targeting antibodies were found to prevent cell-to-cell spike-mediated fusion and neutralize at a post-attachment step. Whether NTD-targeting antibodies neutralize by direct stabilization of spike or indirectly interfere with a putative cell co-receptor or factor that would be required for spike fusogenic activity is currently unknown. However, NTD-targeting antibodies likely impede the normal SARS-CoV-2 Spike function by impeding fusion of virus and host cell membranes via steric hindrance[5] or as previously reported for MERS-CoV NTD-targeting and neutralizing antibody 7D10, by preventing protease cleavage of the Spike[6]. Understanding the mechanism of NTD-based neutralization is an active area of research for multiple groups. We appreciate this comment by the reviewer and added language to page 17 to emphasize these statements.

**3. Many references lack page numbers.**

Thank you, per this reviewer comment and other reviewer comments, we have included additional references and corrected formatting.

*References in relation to above responses to comments:*

1. Liu, L. *et al.* Potent neutralizing antibodies against multiple epitopes on SARS-CoV-2 spike. *Nature* **584**, 450-456 (2020).
2. McCallum, M. *et al.* N-terminal domain antigenic mapping reveals a site of vulnerability for SARS-CoV-2. *Cell* **184**, 2332-2347 e2316 (2021).
3. Corbett, K.S. *et al.* Immune Correlates of Protection by mRNA-1273 Immunization against SARS-CoV-2 Infection in Nonhuman Primates. *bioRxiv* (2021).
4. Suryadevara, N. *et al.* Neutralizing and protective human monoclonal antibodies recognizing the N-terminal domain of the SARS-CoV-2 spike protein. *Cell* **184**, 2316-2331 e2315 (2021).
5. Cerutti, G. *et al.* Potent SARS-CoV-2 neutralizing antibodies directed against spike N-terminal domain target a single supersite. *Cell Host Microbe* **29**, 819-833 e817 (2021).
6. Zhou, H. *et al.* Structural definition of a neutralization epitope on the N-terminal domain of MERS-CoV spike glycoprotein. *Nat Commun* **10**, 3068 (2019).

## Decision Letter, first revision:

**Subject:** Your manuscript, NI-LE32345A

**Message:** Our ref: NI-LE32345A

17th Sep 2021

Dear Dr. Krebs,

Thank you for your patience as we’ve prepared the guidelines for final submission of your Nature Immunology manuscript, "Low-dose in vivo protection and neutralization across SARS-CoV-2 variants by monoclonal antibody combinations" (NI-LE32345A). Please carefully follow the step-by-step instructions provided in the attached file, and add a response in each row of the table to indicate the changes that you have made. Please also check and comment on any additional marked-up edits we have proposed within the text. Ensuring that each point is addressed will help to ensure that your revised manuscript can be swiftly handed over to our production team.

We would like to start working on your revised paper, with all of the requested files and forms, as soon as possible (preferably within two weeks). Please get in contact with us if you anticipate delays.

When you upload your final materials, please include a point-by-point response to any remaining reviewer comments and please make sure to upload your checklist.

If you have not done so already, please alert us to any related manuscripts from your group that are under consideration or in press at other journals, or are being written up for submission to other journals (see: https://www.nature.com/nature-research/editorial-policies/plagiarism#policy-on-duplicate-publication for details).

In recognition of the time and expertise our reviewers provide to Nature Immunology’s editorial process, we would like to formally acknowledge their contribution to the external

peer review of your manuscript entitled "Low-dose in vivo protection and neutralization across SARS-CoV-2 variants by monoclonal antibody combinations". For those reviewers who give their assent, we will be publishing their names alongside the published article.

Nature Immunology offers a Transparent Peer Review option for new original research manuscripts submitted after December 1st, 2019. As part of this initiative, we encourage our authors to support increased transparency into the peer review process by agreeing to have the reviewer comments, author rebuttal letters, and editorial decision letters published as a Supplementary item. When you submit your final files please clearly state in your cover letter whether or not you would like to participate in this initiative. Please note that failure to state your preference will result in delays in accepting your manuscript for publication.

<b>Cover suggestions</b>

As you prepare your final files we encourage you to consider whether you have any images or illustrations that may be appropriate for use on the cover of Nature Immunology.

Covers should be both aesthetically appealing and scientifically relevant, and should be supplied at the best quality available. Due to the prominence of these images, we do not generally select images featuring faces, children, text, graphs, schematic drawings, or collages on our covers.

We accept TIFF, JPEG, PNG or PSD file formats (a layered PSD file would be ideal), and the image should be at least 300ppi resolution (preferably 600-1200 ppi), in CMYK colour mode.

If your image is selected, we may also use it on the journal website as a banner image, and may need to make artistic alterations to fit our journal style.

Please submit your suggestions, clearly labeled, along with your final files. We'll be in touch if more information is needed.

Nature Immunology has now transitioned to a unified Rights Collection system which will allow our Author Services team to quickly and easily collect the rights and permissions required to publish your work. Approximately 10 days after your paper is formally accepted, you will receive an email in providing you with a link to complete the grant of rights. If your paper is eligible for Open Access, our Author Services team will also be in touch regarding any additional information that may be required to arrange payment for your article.

You will not receive your proofs until the publishing agreement has been received through our system.

Please note that <i>Nature Immunology</i> is a Transformative Journal (TJ). Authors may publish their research with us through the traditional subscription access route or make their paper immediately open access through payment of an article-processing charge (APC). Authors will not be required to make a final decision about access to their article until it has been accepted. <a href="https://www.springernature.com/gp/open-

research/transformative-journals">Find out more about Transformative Journals</a>.

If you have any questions about costs, Open Access requirements, or our legal forms, please contact ASJournals@springernature.com.

<B>Authors may need to take specific actions to achieve <a href="https://www.springernature.com/gp/open-research/funding/policy-compliance-faqs">compliance</a> with funder and institutional open access mandates.</b> For submissions from January 2021, if your research is supported by a funder that requires immediate open access (e.g. according to <a href=""https://www.springernature.com/gp/open-research/plan-s-compliance"">Plan S principles</a>) then you should select the gold OA route, and we will direct you to the compliant route where possible. For authors selecting the subscription publication route our standard licensing terms will need to be accepted, including our <a href=""https://www.springernature.com/gp/open-research/policies/journal-policies"">self-archiving policies</a>. Those standard licensing terms will supersede any other terms that the author or any third party may assert apply to any version of the manuscript.

Please use the following link for uploading these materials: [REDACTED]

If you have any further questions, please feel free to contact me.

Best regards,

Elle Morris
Senior Editorial Assistant
Nature Immunology
Phone: 212 726 9207
Fax: 212 696 9752
E-mail: immunology@us.nature.com

On behalf of

Laurie A. Dempsey, Ph.D.
Senior Editor
Nature Immunology
l.dempsey@us.nature.com
ORCID: 0000-0002-3304-796X

Reviewer #2:
Remarks to the Author:
The authors did a great job revising the paper. Nice study!

Reviewer #3:

Remarks to the Author:
The authors have addressed my previous comments. I do not have anything further to add.

Reviewer #4:
Remarks to the Author:
The authors revised well to my points.

**Final Decision Letter:**

**Subject:** Decision on Nature Immunology submission NI-A32345B
**Message:** In reply please quote: NI-A32345B

Dear Dr. Krebs,

I am delighted to accept your manuscript entitled "Low-dose in vivo protection and neutralization across SARS-CoV-2 variants by monoclonal antibody combinations" for publication in an upcoming issue of Nature Immunology.

The manuscript will now be copy-edited and prepared for the printer. Please check your calendar: if you will be unavailable to check the galley for some portion of the next month, we need the contact information of whom will be making corrections in your stead. When you receive your galleys, please examine them carefully to ensure that we have not inadvertently altered the sense of your text.

Acceptance is conditional on the data in the manuscript not being published elsewhere, or announced in the print or electronic media, until the embargo/publication date. These restrictions are not intended to deter you from presenting your data at academic meetings and conferences, but any enquiries from the media about papers not yet scheduled for publication should be referred to us.

Please note that <i>Nature Immunology</i> is a Transformative Journal (TJ). Authors may publish their research with us through the traditional subscription access route or make their paper immediately open access through payment of an article-processing charge (APC). Authors will not be required to make a final decision about access to their article until it has been accepted. <a href="https://www.springernature.com/gp/open-research/transformative-journals">Find out more about Transformative Journals</a>.

<B>Authors may need to take specific actions to achieve <a href="https://www.springernature.com/gp/open-research/funding/policy-compliance-faqs">compliance</a> with funder and institutional open access mandates.</b> For submissions from January 2021, if your research is supported by a funder that requires immediate open access (e.g. according to <a href="https://www.springernature.com/gp/open-research/plan-s-compliance">Plan S principles</a>) then you should select the gold OA route, and we will direct you to the compliant route where possible. For authors selecting the subscription publication route our standard licensing terms will need to be accepted, including our <a href="https://www.springernature.com/gp/open-research/policies/journal-policies">self-archiving policies</a>. Those standard licensing terms will supersede any other terms

that the author or any third party may assert apply to any version of the manuscript.

In approximately 10 business days you will receive an email with a link to choose the appropriate publishing options for your paper and our Author Services team will be in touch regarding any additional information that may be required.

You will not receive your proofs until the publishing agreement has been received through our system.

If you have any questions about our publishing options, costs, Open Access requirements, or our legal forms, please contact ASJournals@springernature.com

Once your manuscript is typeset and you have completed the appropriate grant of rights, you will receive a link to your electronic proof via email with a request to make any corrections within 48 hours. If, when you receive your proof, you cannot meet this deadline, please inform us at rjsproduction@springernature.com immediately. Once your paper has been scheduled for online publication, the Nature press office will be in touch to confirm the details.

Your paper will be published online soon after we receive your corrections and will appear in print in the next available issue. Content is published online weekly on Mondays and Thursdays, and the embargo is set at 16:00 London time (GMT)/11:00 am US Eastern time (EST) on the day of publication. Now is the time to inform your Public Relations or Press Office about your paper, as they might be interested in promoting its publication. This will allow them time to prepare an accurate and satisfactory press release. Include your manuscript tracking number (NI-A32345B) and the name of the journal, which they will need when they contact our office.

About one week before your paper is published online, we shall be distributing a press release to news organizations worldwide, which may very well include details of your work. We are happy for your institution or funding agency to prepare its own press release, but it must mention the embargo date and Nature Immunology. Our Press Office will contact you closer to the time of publication, but if you or your Press Office have any enquiries in the meantime, please contact press@nature.com.

Also, if you have any spectacular or outstanding figures or graphics associated with your manuscript - though not necessarily included with your submission - we'd be delighted to consider them as candidates for our cover. Simply send an electronic version (accompanied by a hard copy) to us with a possible cover caption enclosed.

To assist our authors in disseminating their research to the broader community, our SharedIt initiative provides you with a unique shareable link that will allow anyone (with or without a subscription) to read the published article. Recipients of the link with a subscription will also be able to download and print the PDF.

As soon as your article is published, you will receive an automated email with your shareable link.

You can now use a single sign-on for all your accounts, view the status of all your

manuscript submissions and reviews, access usage statistics for your published articles and download a record of your refereeing activity for the Nature journals.

If you have not already done so, we strongly recommend that you upload the step-by-step protocols used in this manuscript to the Protocol Exchange. Protocol Exchange is an open online resource that allows researchers to share their detailed experimental know-how. All uploaded protocols are made freely available, assigned DOIs for ease of citation and fully searchable through nature.com. Protocols can be linked to any publications in which they are used and will be linked to from your article. You can also establish a dedicated page to collect all your lab Protocols. By uploading your Protocols to Protocol Exchange, you are enabling researchers to more readily reproduce or adapt the methodology you use, as well as increasing the visibility of your protocols and papers. Upload your Protocols at www.nature.com/protocolexchange/. Further information can be found at www.nature.com/protocolexchange/about .

Please note that we encourage the authors to self-archive their manuscript (the accepted version before copy editing) in their institutional repository, and in their funders' archives, six months after publication. Nature Research recognizes the efforts of funding bodies to increase access of the research they fund, and strongly encourages authors to participate in such efforts. For information about our editorial policy, including license agreement and author copyright, please visit www.nature.com/ni/about/ed_policies/index.html

An online order form for reprints of your paper is available at <a href="https://www.nature.com/reprints/author-reprints.html">https://www.nature.com/reprints/author-reprints.html</a>. Please let your coauthors and your institutions' public affairs office know that they are also welcome to order reprints by this method.

Sincerely,

Laurie

Laurie A. Dempsey, Ph.D.
Senior Editor
Nature Immunology
l.dempsey@us.nature.com
ORCID: 0000-0002-3304-796X
